# Transformation of the pectoral girdle in pennaraptorans: critical steps in the formation of the modern avian shoulder joint

**DOI:** 10.7717/peerj.16960

**Published:** 2024-02-29

**Authors:** Qian Wu, Jingmai K. O’Connor, Shiying Wang, Zhonghe Zhou

**Affiliations:** 1 University of Chinese Academy of Sciences, Beijing, China; 2 CAS Center for Excellence in Life and Paleoenvironment, Beijing, China; 3 Key Laboratory of Vertebrate Evolution and Human Origins, Institute of Vertebrate Paleontology and Paleoanthropology, Chinese Academy of Sciences, Beijing, China; 4 Negaunee Integrative Research Center, Field Museum of Natural History, Chicago, IL, United States of America; 5 College of Paleontology, Shenyang Normal University, Shenyang, China

**Keywords:** Flight evolution, Coracoid, Furcula, Scapula, Triosseal canal

## Abstract

Important transformations of the pectoral girdle are related to the appearance of flight capabilities in the Dinosauria. Previous studies on this topic focused mainly on paravians yet recent data suggests flight evolved in dinosaurs several times, including at least once among non-avialan paravians. Thus, to fully explore the evolution of flight-related avian shoulder girdle characteristics, it is necessary to compare morphology more broadly. Here, we present information from pennaraptoran specimens preserving pectoral girdle elements, including all purportedly volant taxa, and extensively compare aspects of the shoulder joint. The results show that many pectoral girdle modifications appear during the evolution from basal pennaraptorans to paravians, including changes in the orientation of the coracoid body and the location of the articulation between the furcula and scapula. These modifications suggest a change in forelimb range of motion preceded the origin of flight in paravians. During the evolution of early avialans, additional flight adaptive transformations occur, such as the separation of the scapula and coracoid and reduction of the articular surface between these two bones, reduction in the angle between these two elements, and elongation of the coracoid. The diversity of coracoid morphologies and types of articulations joining the scapula-coracoid suggest that each early avialan lineage evolved these features in parallel as they independently evolved more refined flight capabilities. In early ornithothoracines, the orientation of the glenoid fossa and location of the acrocoracoid approaches the condition in extant birds, suggesting a greater range of motion in the flight stroke, which may represent the acquisition of improved powered flight capabilities, such as ground take-off. The formation of a new articulation between the coracoid and furcula in the Ornithuromorpha is the last step in the formation of an osseous triosseal canal, which may indicate the complete acquisition of the modern flight apparatus. These morphological transitions equipped birds with a greater range of motion, increased and more efficient muscular output and while at the same time transmitting the increased pressure being generated by ever more powerful flapping movements in such a way as to protect the organs. The driving factors and functional adaptations of many of these transitional morphologies are as yet unclear although ontogenetic transitions in forelimb function observed in extant birds provide an excellent framework through which we can explore the behavior of Mesozoic pennaraptorans.

## Introduction

The pectoral girdle, formed by the scapula, coracoid, and furcula, connects the forelimbs and trunk of tetrapods and provides the attachment point (origin) for the muscles involved in forelimb locomotion; thus, its morphology is crucial for forelimb function (McGonnell, 2001; Benton, 2014). There have been many transformations in the morphology of the pectoral girdle that occurred during the evolution from non-avialan maniraptoran theropod dinosaurs (hereafter simply maniraptorans or theropods) to birds (Avialae) as summarized by previous studies**,** such as the elongation of the coracoid body from a quadrangular (often described as trapezoidal) to strut-like morphology, the separation of the scapula and coracoid from a fused (or connected by long suture prior to fusion) scapulocoracoid to separate bones articulating through a ball and socket joint, the change in the angle demarcated by the scapulocoracoid from obtuse to acute, the orientation of the glenoid fossa from caudoventral to dorsolateral, the elongation of the acrocoracoid process (homologous to “coracoid tubercle” or “biceps tubercle” of theropods) and the rotation of the coracoid body from laterally facing to craniocaudally then ventrally facing (Turner, Makovicky & Norell, 2012; Lü et al., 2016; Wang, Stidham & Zhou, 2018; Novas et al., 2021b). The osseous triosseal canal is an important feature of extant birds that was absent in non-avialan theropods, forming a pulley-like passage that guides the motion of the main muscle responsible for the upstroke, the *M. supracoracoideus* (Mayr, 2021; Wang et al., 2022b). This canal is formed by the acromion process of the scapula, the acrocoracoid process of the coracoid and the epicleideal process of the furcula, mainly through the coracoscapular joint, the scapuloclavicular joint and the acrocoracoclavicular joint (Baumel et al., 1993; Ando & Fukata, 2018). These changes of the scapula-coracoid were accompanied by the appearance of medial fusion of the sternal plates forming a sternum and a decrease in the interclavicular angle of the furcula.

**Figure 1:**
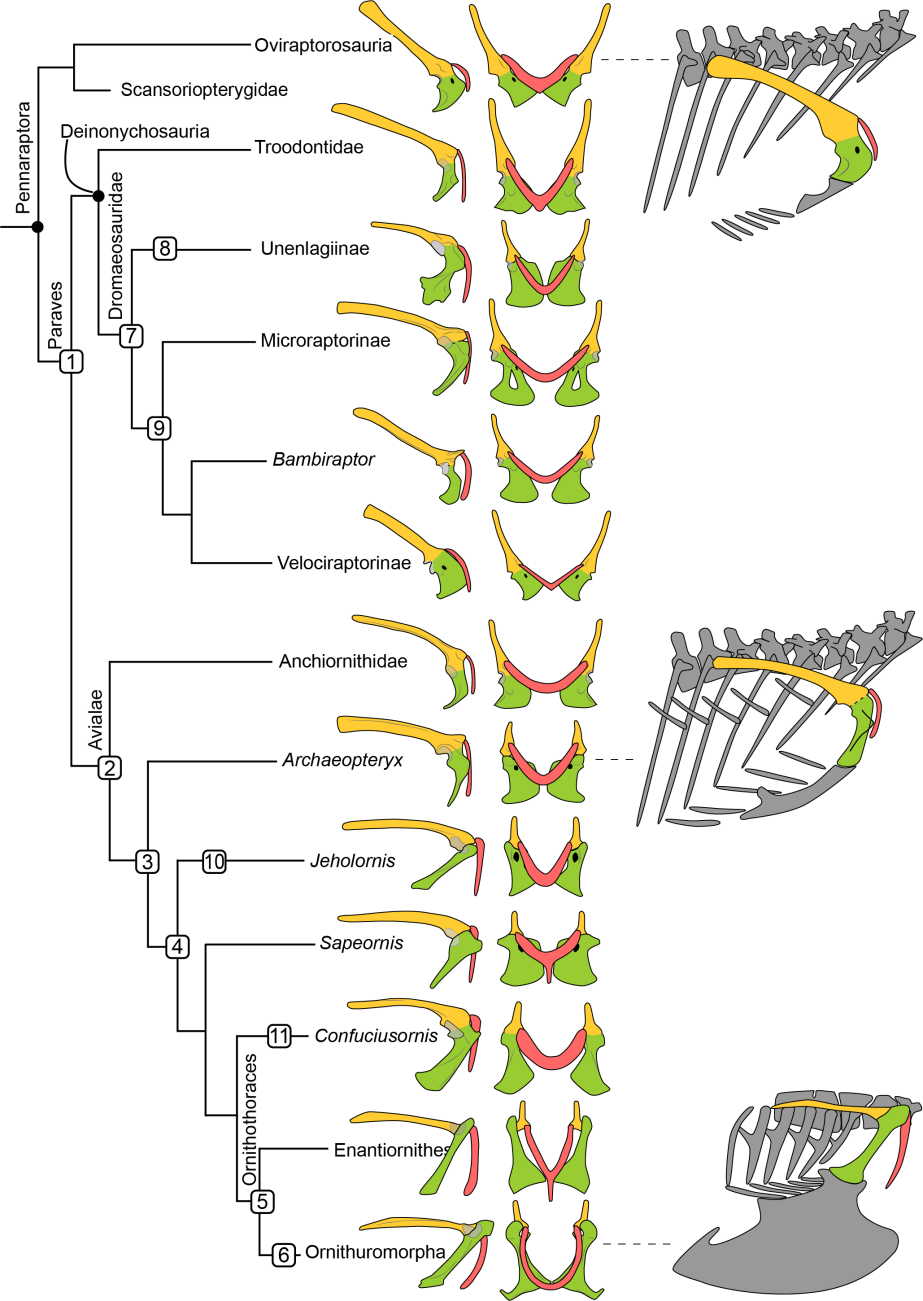
Simplified phylogeny of Pennaraptora with reconstructions of pectoral girdle, the orientation of coracoid body in the skeleton, and the main characters transition. Phylogenetic framework following Pol & Goloboff (2020). Left in lateral view and middle in cranial view. Right in lateral view showing the orientation of the coracoid body. Coracoid facing laterally as in oviraptorosaurs and velociraptorines (top right), facing cranially as in troodontids, potential volant dromaeosaurids, anchiornithids and *Archaeopteryx* (middle right), and facing ventrally as in avialans crownward of *Archaeopteryx* (bottom right). Yellow indicates the scapula, green indicates the coracoid, and red indicates the furcula. Not to scale. 1, rotation of coracoid body, and furcular articulated with cranial tip of acromion process of scapula; 2, localized scapula and coracoid articular surface and lateral oriented glenoid fossa; 3, well develop acromion process of the coracoid; 4 acute angle between scapula and coracoid, and unfused coracoscapular joint; 5, glenoid fossa oriented dorsolaterally, acrocoracoid process dorsally than glenoid fossa, and occurrence of procoracoid process; 6 new joint between coracoid and furcula and the formation of bony closed triosseal canal; 7, lateral oriented glenoid fossa, localized scapula and coracoid articular surface, and well develop acromion process of the coracoid; 8, unfused coracoscapular joint in *Rahonavis*; 9, neck of coracoid body; 10, procoracoid process and strut-like coracoid; 11 strut-like coracoid. 7–9 show the independent evolution of flight adaptive features in several paravian clades.

Traditionally, birds were considered the only volant dinosaurian lineage and research seeking to understand flight related transformations focused on birds and their closest relatives, the Troodontidae and Dromaeosauridae, which all together form the clade Paraves. The Troodontidae and Dromaeosauridae together form the Deinonychosauria, which is commonly resolved as the sister group to Avialae (Turner, Makovicky & Norell, 2012; Sullivan, Xu & O’Connor, 2017). However, recent discoveries suggest that some form of volant ability likely evolved several times independently in the Maniraptora: in the Scansoriopterygidae, Microraptorinae (Dromaeosauridae), Unenlaginae (Dromaeosauridae), and Avialae (Sullivan, Xu & O’Connor, 2017; Pei et al., 2020). All maniraptorans considered to have some volant capabilities belong to the plesiomorphically terrestrial clade Pennaraptora (Fig. 1) (Pol & Goloboff, 2020; Pei et al., 2020). Pennaraptora is a node-based clade, defined as the last common ancestor of *Oviraptor philoceratops* Osborn, 1924, *Deinonychus antirrhopus* Ostrom, 1969, and *Passer domesticus* Linnaeus, 1758, and all its descendants (Foth, Tischlinger & Rauhut, 2014). The Pennaraptora consists of Paraves together with the Oviraptorosauria and Scansoriopterygidae, the latter commonly regarded as a basal lineage of the Oviraptorosauria (Gianechini et al., 2018; Pittman & Xu, 2020). Pennaceous feathers, which are crucial for flight in at least the Microraptorinae and Avialae, are only found in pennaraptorans (Lefèvre et al., 2020; Pittman & Xu, 2020).

In light of the recent discovery of volant abilities in scansoriopterygids, to fully understand the evolution of flight-related pectoral girdle characters we must explore the pectoral girdle morphology beyond Paraves to include a wider range of taxa across all of Pennaraptora (Xu et al., 2015; Wang et al., 2019). Furthermore, the continuous discovery of new taxa and specimens means that summaries of morphology and disparity pertaining to particular anatomical regions need to be regularly updated, especially in light of the increasing availability of computed tomographic (CT) data that reveals anatomical features in 3D and greater clarity. For example, reduction of the angle between the scapula and coracoid was once thought to be an exclusively avialan feature but recently was reported to be also less than 90° in the troodontid *Liaoningvenator* (Shen et al., 2017). In order to better understand flight related morphological transformations of the shoulder girdle features, here we utilize available data concerning the morphology of the pectoral girdle across pennaraptorans to summarize the variation, make comparisons between clades and taxa, and discuss the possible relationship between these transformations and changes in forelimb function as it pertains to the evolution of flight. This study will provide detailed information about the morphological and functional comparation of the shoulder girdle of pennaraptors for paleontologist who are interested in the evolution of pennaraptors and the origin of flight in early birds.

## Survey Methodology

We collected morphology information of pennaraptor specimens reported in literatures and focus on those preserved shoulder girdle elements. The characters of the shoulder girdle of different taxa of pennaraptors are re-evaluated and compared based on text and figures from the origin literatures, and listed in Table S1. We further listed those characters that are considered to be closely related to flight evolution in Table 1.

**Table 1:** Overview of flight-related pectoral girdle features in pennaraptorans and the inferred flight capacities. Features similar to condition of Ornithuromorpha are highlighted in bold. GFO, glenoid fossa orientation (0: caudoventral; 1: lateral; 2: dorsolateral); CSJ, coracoscapular joint (0: long synchondrosis fused in adult; 1: localized articular surface fused in adult; 2: localized articular surface unfused but tightly articulated; 3: localized articular surface fused during early ontogeny; 4: localized articular surface of flexible joint); PAR, acromion process of the scapula (0: caudal to coracoscapular articular surface; 1: dorsally at the same level as the coracoscapular articular surface; 2: cranially over the coracoscapular articular surface); SCJ, scapuloclavicular joint (0: along the dorsal edge of the scapula; 1: at the cranial tip of the acromion process of the scapula); PAC, acrocoracoid process (0: ventral to the coracoid foramen; 1: same level as the coracoid foramen; 2: close to the glenoid fossa; 3: same level as the glenoid fossa; 4 dorsally over the glenoid fossa); ACJ, acrocoracoid process of the coracoid articulate with the furcula form the acrocoracoclavicular joint (0: no; 1: yes); PPC, coracoid with procoracoid process (0: no; 1: yes); SCA, scapula-coracoid angle (0: <110°; 1: 90–100°; 2: <90°); CRS, coracoid shape (0: trapezoidal; 1: mediolaterally narrow forming a distinct neck near the scapular articular surface; 2: strut-like); CBO, coracoid body orientation (0: mediolateral; 1: dorsoventral); IFC, inferred flight capacity (0: terrestrial; 1: potential volant and gliding flight; 2: weak powered flight; 3: intermittent powered flight, 4 continuous powered flight).

	GFO	CSJ	PAR	SCJ	PAC	ACJ	PPC	SCA	CRS	CBO	IFC
Early pennaraptorans	0	0	0	0	0	0	0	0	0	0	0
Troodontidae	0	0	1	**1**	1	0	0	1	0	**1**	0
*Buitreraptor*	1	1	**2**	**1**	2	0	0	1	0	**1**	1
*Rahonavis*	1	2	**2**	**1**	?	?	?	?	?	?	2
Microraptorinae	1	1	**2**	**1**	2	0	0	1	1	**1**	1
*Bambiraptor*	1	1	**2**	**1**	2	0	0	1	1	**1**	1
Velociraptorinae	0	0	1	**1**	0	0	0	0	0	0	0
Anchiornithidae	1	1	0	**1**	2	0	0	**2**	0	**1**	1
*Archaeopteryx*	1	1	**2**	**1**	2	0	0	1	0	**1**	2
*Jeholornis*	1	2	**2**	**1**	3	0	**1**	**2**	**2**	**1**	2
*Sapeornis*	1	2	**2**	**1**	3	0	0	**2**	0	**1**	2
*Confuciusornis*	1	3	**2**	**1**	3	0	0	**2**	**2**	**1**	2
Enantiornithes	**2**	**4**	**2**	**1**	**4**	0	0	**2**	**2**	**1**	3
Ornithuromorpha	**2**	**4**	**2**	**1**	**4**	**1**	**1**	**2**	**2**	**1**	**4**

### Phylogenetic framework

We follow the recent phylogeny by Pol & Goloboff (2020), in which Pennaraptora is formed by Oviraptorosauria, Scansoriopterygidae, Dromaeosauridae, Troodontidae and Avialae (Fig. 1)**.** ‘*Scansoriopteryx’* is interpreted as a junior synonym of *Epidendrosaurus* (Padian, 2004; Feduccia, Lingham-Soliar & Hinchliffe, 2005). *Ingenia yanshini* (Barsbold, 1981) was renamed as *Ajancingenia yanshini* (Easter, 2013), and later the genus name *‘Ajancingenia’* is considered to be synonym of *Heyuannia* (Funston et al., 2018), which is also accepted here. Anchiornithidae is interpreted as early diverging members of Avialae (Pol & Goloboff, 2020). ‘*Aurornis’* is considered a junior synonym of *Anchiornis* (Pei et al., 2017). As part of the extant phylogenetic bracket, additional comparative morphological data comes from extant crocodilians, which like dinosaurs are archosaurian reptiles.

### Nomenclature

The anatomical nomenclature primarily follows Baumel et al. (1993), and osteological structures are described using the English equivalents of Latin terms. The “coracoid tubercle” (or coracoid tuber) or “biceps tubercle” in theropods is considered homologous with the modern avian “acrocoracoid process” (Ostrom, 1976). For convenience, this feature is referred to as the “acrocoracoid process” throughout; similarly, the posteroventral process of the coracoid in dinosaurs is homologous with and here referred to as the sternolateral process, as it is called in birds.

### Position of the pectoral girdle

The position and orientation of the pectoral girdle is an important basis for evaluating the orientation of the glenoid fossa. In extant volant birds, the scapula is located at the dorsal side of the thoracic cage and parallel to the vertebral column, and the orientation of the coracoid is described as dorsoventral in long axis (the external surface facing ventrally) (Fig. S1). The scapula of flightless extant common ostrich is located at the lateral side of the thoracic cage and the external surface of the coracoid body facing cranially (Fig. S2). Here the position of the pectoral girdles of fossil taxa are evaluated based primarily on 3-dimentional (3D), articulated preserved specimens of early diverged taxa of each clade. The pectoral girdle of oviraptorosaurs is place on the lateral side of the thoracic cage with the scapula oblique with the vertebral column, and the external surface of the coracoid body craniolateral facing. The pectoral girdle of troodontids, non-velociraptorines dromaeosaurids and anchiornithids are located similarly as that of extant common ostrich. The scapula of basal birds is parallel to the vertebral column as in extant volant birds.

### Institutional abbreviations

AMNH, American Museum of Natural History, New York, USA; BMNHC, Beijing Museum of Natural History, Beijing, China; BPM, Beipiao Paleontological Museum, Liaoning, China; CAGS, China Academy of Geological Sciences, Beijing, China; DNHM, Dalian Natural History Museum, Dalian, China; GMNH, Ganzhou Museum of Natural History, Ganzhou City, Jiangxi Province, China; GMV, Geological Museum of China, Beijing, China; GSGM, Gansu Geological Museum, Lanzhou, China; HGM, Henan Geological Museum, Henan, China; HYMV, Heyuan Museum, Guangdong, China; IGM, Institute of Geology, Ulaan Baatar, Mongolia; IVPP, Institute of Vertebrate Paleontology and Paleoanthropology, Beijing, China; MACN, Sección Paleontología de Vertebrados, Museo Argentino de Ciencias Naturales, Buenos Aires, Argentina; MCCM, Museo de Cuenca, Cuenca, Spain; MCF, Museo Carmen Funes, Plaza Huincul, Argentina; MCPA, Museo Provincial “Carlos Ameghino,” Cipolletti, Patagonia, Argentina; MNU, Mongolian National University, Mongolia; MPC, Institute of Paleontology and Geology, Mongolian Academy of Sciences, Ulaan baatar, Mongolia; MPC-NEE, Nemegt Educational Expedition field number, specimens housed at the Institute of Paleontology and Geology, Mongolian Academy of Sciences, Ulaanbaatar, Mongolia; MPD, Mongolia Palaeontological Centre, Mongolia; MTM, Hungarian Natural History Museum, Budapest, Hungary; LPM, Liaoning Paleontology Museum,. Shenyang Normal University, Shenyang, China; PIN, Paleontological Institute, Russian Academy of Sciences, Moscow, Russia; PMOL, Palaeontological Museum of Liaoning, China; PVL, Paleontologia de Vertebrados M. Lillo, Universidad Nacional de Tucumán, Tucmán, Argentina; SMM, Sternberg Memorial Museum, Kansas, USA; STM, Shandong Tianyu Museum of Nature, Shandong, China; TMP, Royal Tyrrell Museum of Palaeontology, Drumheller, Alberta, Canada; UA, Université d’Antananarivo, Antananarivo, Madagascar; YGFP, Yizhou Fossil and Geology Park, Yizhou, China; YPM, Yale Peabody Museum, New Haven, Connecticut, USA.

## Compare of Pennaraptoran Pectoral Girdle

### Oviraptorosauria

In oviraptorosaurs, the pectoral girdle are located on the lateral side of the thoracic cage, with the scapular blade oriented laterally forming an obvious angle with the vertebral column, and the external surface of the coracoid body oriented craniolaterally, as shown in the early diverging Early Cretaceous *Caudipteryx* IVPP V12430 and BPM 0001 (Zhou et al., 2000), and *Similicaudipteryx* STM 22-6 (Xu, Zheng & You, 2010), and the 3D preserved, nearly complete and articulated specimen *Khaan* IGM 100/1002 (Balanoff & Norell, 2012). The scapula and coracoid are fused or tightly articulated through a long synchondrosis (Osmólska, Currie & Barsbold, 2004), with the angle between the scapula and coracoid exceeding 90° as observed in *Tongtianlong* DYM-2013-8, *Heyuannia* HYMV1-2 and 1-5, and *Avimimus* PIN 3907-1 (Table S1) (Kurzanov, 1981), and the scapulocoracoid flexion within the coracoid (Paul, 2002; Novas et al., 2021b). Previously, this articulation was described as a “suture” in some specimens (Makovicky & Sues, 1998; He, Wang & Zhou, 2008; Funston et al., 2018). However, this is a misapplication of the term. In anatomy, a “suture” indicates the rigid fibrous joint between membranous bones, such as the sutures in the skull (Hall, 2005; Ding & Liu, 2018). Since both the scapula and coracoid are endochondral bones, the appropriate term for such a tight articulation through cartilage should be “synchondrosis”, as in extant crocodilians (Brochu, 1995).

Most oviraptorosaur specimens identified as adults have fused scapulocoracoids (Table S1), except for one specimen of *Caudipteryx* IVPP V12430 (Zhou & Wang, 2000) which is a small individual and may in fact be immature. The scapula and coracoid are unfused (including fully separated and joined through synchondrosis) in 16 out of 27 reported oviraptorosaur species (Table S1), all of which are based on material that is inferred to be ontogenetically immature (juvenile or subadult) (Table S1), as in *Yulong* HGM 41HIII-0107 (Lü et al., 2013a), *Rinchenia* MPC-D 100/32A (Funston et al., 2018) and *Gobiraptor* MPC-D 102/111 (Lee et al., 2019). This evidence strongly suggests that fusion of the synchondrosis joint of the scapulocoracoid is related to ontogeny in oviraptorosaurs as in extant crocodilians (Brochu, 1995), and that the scapulocoracoid is fused in adults (Funston et al., 2021). The glenoid fossa of oviraptorosaurs is described as caudoventrally oriented (Osmólska, Currie & Barsbold, 2004), as in *Oksoko* MPC-C 102/110 (Funston, 2020), *Avimimus* MPC-NEE.2016-257 (Funston et al., 2018), *Anzu* CM 78000 (Lamanna et al., 2014) (Table S1), but the glenoid was described as laterally oriented in some Late Cretaceous taxa, such as *Heyuannia* HYMV1-2 (Lü, 2003;Lü, Huang & Qiu, 2005), *Khaan* IGM 100/1002 (Balanoff & Norell, 2012), and *Apatoraptor* TMP 1993.051.0001 (Funston & Currie, 2016).

**Figure 2:**
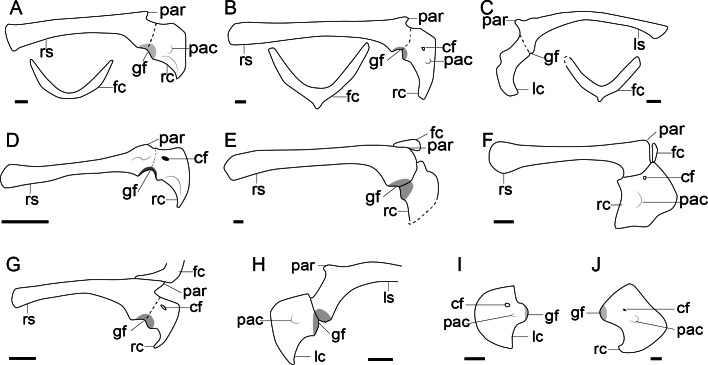
Comparison of the pectoral girdle of oviraptorosaurs. (A) *Khaan* based on IGM 100/1002 and IGM 100/1127 (Balanoff & Norell, 2012); (B) *Oksoko* based on MPC-C 102/110 (Funston et al., 2020); (C) *Oviraptor* based on IGM 100/36 and 100/42 modified after Barsbold (1983); (D) *Heyuannia* based on MPC-D 100/30 modified after Osmólska, Currie & Barsbold (2004); (E) *Rinchenia* based on MPC-D 100/32-A (Funston et al., 2018); (F) *Caudipteryx* based on BPM 001 modified after (Zhou et al., 2000); (G) *Nankang* based on GMNH F10003 (Lü et al., 2013b); (H) *Elmisaurus* based on MPC-D 102/113 (Funston et al., 2021); (I) *Microvenator celer* based on AMNH 3041 modified after Makovicky & Sues (1998); (J) *Chirostenotes* based on TMP 1979. 020.0001 (Funston, 2020). Reconstructed portion marked with dash line; gray color indicates the articular surface for humerus when identical from the specimen during reconstruction. Scapula and coracoid in lateral view, and furcula in cranial view. Abbreviations: cf, coracoid foramen; fc, furcula; gf, glenoid fossa; lc, left coracoid, ls, left scapula; rc, right coracoid; rs, right scapula; pac, acrocoracoid process; par, acromion process; ppc, procoracoid process. Scale bar = 1 cm.

The 2D preservation of *Apatoraptor* makes interpretations equivocal for this taxon. The figure of *Heyuannia* HYMV1-2 in the original publication does not show the glenoid fossa since it is shield by the humerus, and in *Heyuannia* MPC-D 100/30 the glenoid fossa appears to be caudoventrally oriented (Fig. 2D). Although the glenoid fossa of *Khaan* is indeed oriented more laterally than other oviraptorosaurs, being ventrolaterally rather than caudoventrally oriented (Fig. 2A) (Balanoff & Norell, 2012), it is not as laterally as in the extant ostrich, since the glenoid located on the caudal.

In light of such equivocal evidence, we prefer to be conservative about the orientation of glenoid fossa in oviraptorosaurs in which preservation is not clear and consider that the glenoid is caudoventrally oriented in the majority of taxa with the possible exception of the Late Cretaceous oviraptorid *Khaan*, in which changes in orientation are clearly apomorphic. Clearly, caudoventral orientation of the glenoid represents the plesiomorphic condition although a shift in the orientation of this articular surface may have evolved independently in some late- diverging lineages by the Late Cretaceous (Funston et al., 2020).

The acromion process of the scapula is well-developed and inflected craniolaterally in oviraptorosaurs (Fig. 2), as in *Khaan* IGM 100/1002 (Balanoff & Norell, 2012), *Oksoko* MPC-D 102/110 (Funston et al., 2020) and *Heyuannia* MPC-D 100/30 (Osmólska, Currie & Barsbold, 2004), providing a sizeable articular surface along the dorsal edge of the scapula for the furcula (Balanoff & Norell, 2012). The joint between the acromion and furcula is clearly visible in *Rinchenia* and *Nankangia*, whose acromion and furcula are preserved in articulation (Fig. 2) (Lü et al., 2013b; Funston et al., 2018). *Caudipteryx* was previously described as without a prominent acromion process (Zhou et al., 2000), but re-examination reveals that its acromion process is similar to other oviraptorosaurs (Fig. 2F). The acromion process is close to but caudal to the articular surface of scapula and coracoid in oviraptorosaurs (Fig. 2), with the exception of the diverged Late Cretaceous caenagnathids *Elmisaurus* which has an acromion located a significant distance caudal to the scapula and coracoid articulation (Funston et al., 2021). Accordingly the acromion and furcula articulation may also be more caudally located in *Elmisaurus*.

The external surface of the coracoid body of oviraptorosaurs is craniolaterally oriented and trapezoidal, with relatively short and straight cranial and omal margins, a concave caudal margin and a convex sternal margin (Figs. 2, S3) (Osmólska, Currie & Barsbold, 2004). A prominent acrocoracoid process (coracoid tubercle) is located on the craniolateral surface of the coracoid (Fig. 2) (Osmólska, Currie & Barsbold, 2004), generally located below the coracoid foramen, if the latter is present, as in *Heyuannia* MPC-D 100/30 (Osmólska, Currie & Barsbold, 2004), *Oksoko* MPC-D 100/30 (Funston et al., 2020), *Chirostenotes* TMP 1979.020.0001 (Funston, 2020) and *Microvenator* AMNH 3041 (Makovicky & Sues, 1998). The sternal end of the coracoid is craniocaudally expanded with a sternolateral process, and articulated with the transverse groove on the craniodorsal edge of the sternum in oviraptorids (Osmólska, Currie & Barsbold, 2004). While in *Caudipteryx* the sternal plates are oval and separate, and as preserved they lack sternal processes, as in BPM001 and IVPP V 12344 (Ji et al., 1998; Zhou et al., 2000; Zhou & Wang, 2000). The coracoid and sternal plates of *Caudipteryx* may connect in a different way from that of oviraptorids, like by soft tissue, which needs further investigation on specimens to reveal.

The coracoid in *Avimimus* was originally described as elongated based on specimen PIN #3907/1 (Kurzanov, 1981) and later described as triangular (MPC-NEE.2016-257) (Funston et al., 2018). Funston and coauthors states that the triangular shape of the coracoid in *Avimimus* is due to the enlargement of the cranioventral part (Funston et al., 2018). However, given the morphological difference between the coracoid in these two specimens, and the poor preservation of *Avimimus* specimens, it cannot be ruled out that this unusual morphology is a preservational artifact.

### Scansoriopterygidae

Scansoriopterygids are hypothesized to have a unique form of gliding flight utilizing a forelimb membrane supported by the apomorphic elongate third digit and styliform process (Xu et al., 2015; Wang et al., 2019; Dececchi et al., 2020). There is no consensus as to their phylogenetic position. They have been regarded as members of the Oviraptorosauria (Agnolín & Novas, 2013; O’Connor & Sullivan, 2014; Brusatte et al., 2014; Pittman & Xu, 2020; Pei et al., 2020), early-diverging paravians (O’Connor & Sullivan, 2014; Wang et al., 2019), or even avialans (Senter, 2007; Zhang et al., 2008; Xu et al., 2011). Unlike the abundant remains available for other pennaraptoran clades, scansoriopterygids are exceedingly rare, with only four species, *Epidendrosaurus* (Zhang et al., 2002; Czerkas & Yuan, 2002), *Epidexipteryx* (Zhang et al., 2008), *Yi* (Xu et al., 2015) and *Ambopteryx* (Wang et al., 2019), each known from a single specimen with the exception of *Epidendrosaurus* (known from two immature specimens). The scansoriopterygid affinity of *Zhongornis* is controversial (Gao et al., 2008; O’Connor & Sullivan, 2014), hence it will not be considered here.

**Figure 3:**
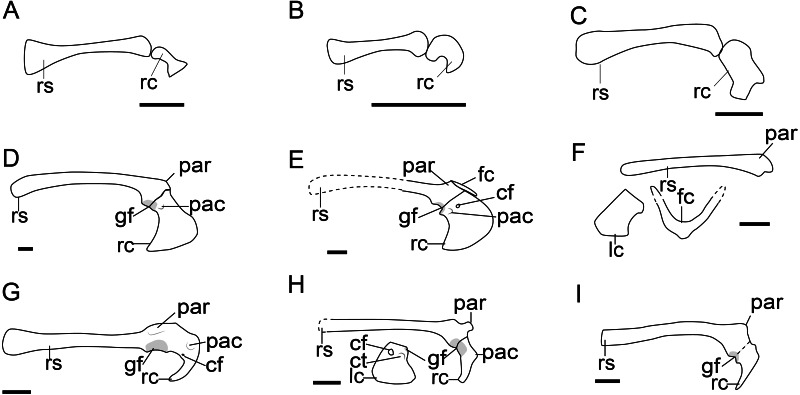
Comparison of the pectoral girdle of Scansoriopterygidae and Troodontidae. (A) *Ambopteryx* based on IVPP V24192 modified from Wang et al. (2019); (B) *Epidendrosaurus* based on IVPP V12653 modified from Zhang et al. (2002); (C) *Epidexipteryx* based on IVPP V15471 modified from Zhang et al. (2008); (D) *Gobivenator* based on MPC-D 100/86 (Tsuihiji et al., 2014); (E) *Sinornithoides* based on IVPP V9612, modified from Currie & Dong (2001); (F) *Mei* based on IVPP V12733 (Xu & Norell, 2004); (G) *Pneumatoraptor* based on MTM V 2008. 38.1 (Ősi, Apesteguía & Kowalewski, 2010); (H) *Sinovenator* based on IVPP V12615 (Xu et al., 2002); (I) *Liaoningvenator* based on DNHM D3012 (Shen et al., 2017). Reconstructed portion marked with dash line; gray color indicates the articular surface for humerus when identical from the specimen during reconstruction. Right scapulocoracoid in lateral view, left coracoid and furcula (F) in cranial view. Abbreviations: cf, coracoid foramen; fc, furcula; gf, glenoid fossa; lc, left coracoid, ls, left scapula; rc, right coracoid; rs, right scapula; pac, acrocoracoid process; par, acromion process; ppc, procoracoid process. Scale bar = 1 cm.

The scapula and coracoid are unfused in *Epidendrosaurus* IVPP V12653, *Epidexipteryx* IVPP V15471, and *Ambopteryx* IVPP V24192 (Fig. 3, Table S1), but all these specimens represent individuals that are interpreted as juvenile or subadult (Czerkas & Yuan, 2002; Zhang et al., 2002; Zhang et al., 2008; Wang et al., 2019). The holotype of *Yi* is considered to be an adult, and only the scapula is preserved (Xu et al., 2015). The proximal end of the scapula is complete, suggesting the scapula and coracoid are not fused in this *Yi qi* specimen. We hope that the discovery of additional specimens will further support this speculation.

Although described simply as subquadrilateral (Czerkas & Yuan, 2002; Zhang et al., 2008), the scansoriopterygid coracoid varies in shape. The coracoids of the young juvenile represented by *Epidendrosaurus* IVPP V12653 and CAGS02-IG-gausa-l/DM 607 are unusual small. *Ambopteryx* IVPP V24192 has a trapezoidal coracoid with concave lateral margin (Fig. 3A) (Wang et al., 2019), while the coracoid of *Epidexipteryx* IVPP V15471 is polygonal (Fig. 3C) (Zhang et al., 2008). Given the poor preservation of these specimens, it is impossible to determine from the currently available evidence whether or not these morphological differences are genuine. Due to the two-dimensional preservation of available specimens, there is insufficient data to make further comparison of the shoulder girdle joint in this clade.

### Troodontidae

The scapula of troodontids is located similarly as that of extant flightless ratite (Figs. 1, S2), with the coracoid body craniocaudal facing, as in the articulated specimens Early Cretaceous *Liaoningvenator* DNHM D3012 (Shen et al., 2017), *Mei* IVPP V 12733 (Xu & Norell, 2004), *Jinfengopteryx* CAGS-IG-04-080 (Ji et al., 2005), *Jianianhualong* DLXH 1218 (Xu, Qin & Palasiatica, 2017), as well as the Late Cretaceous *Gobivenator* MPC-D 100/86 (Tsuihiji et al., 2014). As a result of the cranial rotation of the coracoid, when the tightly articulated or fused scapulocoracoid is preserved in lateral view, only the narrow lateral margin of the coracoid is visible, showing a cranially projecting acrocoracoid process that forms an L-shaped scapulocoracoid, as in *Sinovenator* IVPP V12615 (Xu et al., 2002), *Liaoningvenator* DNHM D 3012 (Shen et al., 2017), and *Pneumatoraptor* MTM 2008.38.1 (Ősi, Apesteguía & Kowalewski, 2010). In these specimens, the scapula is oblique to the vertebral column except in *Mei*, whose scapula almost parallel to the vertebral column (Xu & Norell, 2004; Gao et al., 2012).

In troodontids the scapula and coracoid are connected through a long synchondrosis (Figs. 3D–3I), which may become fused in adults, as in *Jinfengopteryx* CAGS-IG-04-0801 (Ji et al., 2005) and *Pneumatoraptor* V.2008.38.1 (Ősi, Apesteguía & Kowalewski, 2010). These two elements are separate in juvenile specimens, *e.g.*, *Mei* IVPP V12733 (Xu & Norell, 2004). This suggests the fusion of the scapula and coracoid in troodontids is also influenced by ontogeny, as in oviraptorosaurs. The angle between the scapula and coracoid are equal to or slightly less than 90° (Figs. 3D–3I and Table S1), forming a smaller angle than observed in oviraptorosaurs. Although the glenoid orientation is described as caudally or ventrally in the 3D preserved articulated specimens *Liaoningvenator* DNHM D3012 (Shen et al., 2017), *Mei* DNHM D2514 (Gao et al., 2012), and *Sinornithoides* IVPP V9612 (Currie & Dong, 2001) , it is also weakly oriented laterally in some species, *e.g.*, *Sinovenator* (Wang et al., 2022b) and *Gobivenator* MPC-D 100/86 (Tsuihiji et al., 2014). As in oviraptorosaurs, it is unclear to what degree this variation is an artifact of preservation.

The acromion of troodontids is well-developed (Fig. 3). In *Gobivenator* (Tsuihiji et al., 2014) and *Sinovenator* (Wang et al., 2022b), the acromion is located dorsally at the same level as the articular surface for the coracoid, which is cranial to the position of acromion in oviraptorosaurs (Fig. 2). The acromion and furcula of *Sinornithoide* s IVPP V9612 are preserved in articulation (Currie & Dong, 2001), which shows that the articular surface for the furcula is at the cranial tip of the acromion (Fig. 3), whereas in oviraptorosaurs it is located on the dorsal edge (Figs. 1, 2).

Different from the lateral facing coracoid in oviraptorosaurs, *Mei* IVPP V12733 and DNHM D2514, which are three-dimensionally preserved in articulation, and *Sinovenator* (Wang et al., 2022b) show that the body of the coracoid is rotated so that it is facing cranial (Xu & Norell, 2004; Gao et al., 2012). As in oviraptorosaurs, in troodontids the coracoid body is trapezoidal in cranial view, with the omal and medial margins relatively straight, a lateral margin concave, and convex sternal margin (Figs. 3D–3I), and well-developed sternolateral and acrocoracoid processes, *e.g.*, in *Mei* IVPP V12733 (Xu & Norell, 2004; Gao et al., 2012), *Gobivenator* MPC-D 100/86 (Tsuihiji et al., 2014), and *Sinovenator* IVPP V12615 (Wang et al., 2022b). The coracoid of *Sinovenator* IVPP V12615 possesses an acrocoracoid process nearly at the same level as the coracoid foramen (Wang et al., 2022b), more dorsally located than that in the oviraptorosaurs.

### Dromaeosauridae

**Figure 4:**
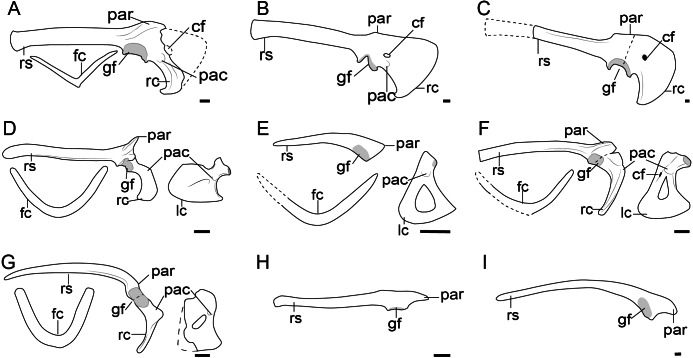
Comparison of the pectoral girdle of Dromaeosauridae. (A) *Velociraptor* based on IGM 100/986 and 976 modified after Norell & Makovicky, 1999; (B) *Deinonychus* based on AMNH 3015 modified after Ostrom (1974); (C) *Achillobator* based on MNU FR-15 modified after Perle, Norell & Clark (1999); (D) *Bambiraptor* based on AMNH FR 30554 modified from Burnham (2004); (E) *Wulong* based on D2933 modified from Poust et al. (2020); (F) *Sinornithosaurus* based on IVPP V12811 modified from Xu, Wang & Wu (1999); (G) *Buitreraptor* based on MPCN-PV-598 modified after Novas et al. (2018); (H) *Rahonavis* based on UA 8656 modified after Forster et al. (2020); (I) *Unenlagia* based on MCF PVPH 78 modified from Novas & Puerta (1997). Reconstructed portion marked with dash line; gray color indicate the articular surface for humerus when identical from specimen during reconstruction. Right scapulocoracoid in lateral view, left coracoid and furcula in cranial view. Abbreviations: cf, coracoid foramen; fc, furcula; gf, glenoid fossa; lc, left coracoid, ls, left scapula; rc, right coracoid; rs, right scapula; pac, acrocoracoid process; par, acromion process; ppc, procoracoid process. Scale bar = 1 cm.

In the phylogeny framework we follow, Dromaeosauridae specimens preserved with pectoral girdle typically consist of several groups, including Unenlagiinae, Microraptorinae, Velociraptorinae and Dromaeosaurinae, as well as some species that have not been assigned to any particular subfamily. The scapula of dromaeosaurids set at the lateral side of the thoracic cage (Senter, 2006), oblique to the vertebral column, as shown in the articulated specimens of Early Cretaceous *Microraptor* IVPP V 13352 (Xu et al., 2003) and *Zhenyuanlong* JPM-0008 (Lü et al., 2016), and 3D preserved articulated specimens of the Late Cretaceous *Linheraptor* (IVPP V 16923) (Xu et al., 2010) and *Velociraptor* IGM 100/976 (Senter, 2006). The fused scapulocoracoids of *Velociraptor* IGM 100/986, *Deinonychus* AMNH 3015, *Achillobator* MNU FR-15 are preserved quite similar to that of oviraptorosaurs (Figs. 4A–4C) (Ostrom, 1974; Norell & Makovicky, 1999; Perle, Norell & Clark, 1999), but differ from troodontids (Figs. 3D–3I), indicating that the coracoid these dromaeosaurids may still be craniolaterally facing as in oviraptorosaurs. On the contrary, the fused scapulocoracoid of microraptorines show L-shape in lateral view like that of troodontids, as in *Microraptor* IVPP V 13352 (Xu et al., 2003) and LVH 0026 (Gong et al., 2012), *Changyuraptor* HG B016 (Han et al., 2014), so as the coracoid of Late Cretaceous *Buitreraptor* MPCN-PV-598 (Novas et al., 2018) indicating the cranial orientation of the coracoid in microraptorines and unenlagiines.

The angle between the scapula and coracoid in velociraptorines and dromaeosaurines is also obtuse (from 120° to 135°) as in oviraptorosaurs (Fig. 2; Figs. 4A–4C). The glenoid fossa of *Velociraptor* shows a similar caudoventral orientation to that of more basal theropods (Senter, 2006), whose osseous floor of the glenoid fossa is mostly visible in caudal view in specimen IGM 100/986 (Norell & Makovicky, 1999). The boomerang-shaped furcula of the *Velociraptor* IGM 100/976 was preserved attach with the well-developed acromion, as in troodontids(Norell & Makovicky, 1999).

As in oviraptorosaurs, the coracoid of velociraptorines is trapezoidal in lateral view, with the cranial and sternal margins slightly convex, and the caudal margin concave with a hooked sternal lateral process in *Velociraptor* IGM 100/986 and *Deinonychus* AMNH 3015 (Figs. 4A, 4B) (Ostrom, 1974; Norell & Makovicky, 1999). The acrocoracoid process is not very pronounced and is located near the caudal margin of the coracoid in *Velociraptor* IGM 100/986 and *Deinonychus* AMNH 3015 (Fig. 4A, 4B) (Ostrom, 1974; Norell & Makovicky, 1999).

In contrast, microraptorines and unenlagiines show significant changes in the morphology of the pectoral girdle (Fig. 4). In species of the Microraptorinae, the scapulocoracoid is fused in adult specimens of *Microraptor*, *e.g.*, CAGS 20-7-004 (Hwang et al., 2002), but unfused in the juvenile holotype of *Wulong* DNHM D2933 (Poust et al., 2020). This pattern is consistent with the oviraptorosaurs and troodontids. Although the scapula and coracoid are unfused in *Sinornithosaurus* IVPP V12811, whose relatively large size suggests it may be an adult individual (Xu, Wang & Wu, 1999), its scapula and coracoid are tightly articulated, and preserved in a state similar to *Microraptor* IVPP V13352 (Xu et al., 2003), indicating the individual may have died just prior to fusion. In *Buitreraptor* MCPA 245 and MPCN-PV-598, the scapula and coracoid are tightly articulated as in *Sinornithosaurus* IVPP V12811, with marked rugosities that indicate ongoing fusion on the coracoidal articular surface (Brochu, 1995; Parsons & Parsons, 2015; Gianechini et al., 2018; Novas et al., 2018). A similarly rugose articular surface is also observed on the scapula of *Unenlagia* MCF PVPH 78 (Novas et al., 2021a). The only exception is the smooth coracoid facet on the scapula of *Rahonavis* UA 8656 (Forster et al., 2020), which suggests a derived avian-like coracoscapular joint that is apomorphic in *Rahonavis* and may be related to flight capabilities in this taxon.

In these dromaeosaurines and unenlagiines, as well as the *Bambiraptor* AMNH FR 30554, the articular surface between the scapula and coracoid is shortened from the long condition in oviraptorosaurs, troodontids, and velociraptorine dromaeosaurids, to a more localized facet, as in, *Rahonavis* UA 8656 (Forster et al., 1998) and *Wulong* D2933 (Poust et al., 2020). When preserved in lateral view, only the narrow lateral margin of the cranially facing coracoid is visible, as in *Buitreraptor* (MPCN-PV-598), *Microraptor* IVPP V13353 and *Sinornithosaurus* IVPP V12811 (Fig. 4) (Xu, Wang & Wu, 1999; Xu et al., 2003; Novas et al., 2018), which confirms the cranial rotation of the coracoid body as in troodontids. The angle between the scapula and coracoid ranges from 90 to 100°, smaller than in oviraptorosaurs and non-volant dromaeosaurids (Fig. 2; Figs. 4A–4C). The glenoid fossa of microraptorines, unenlagiines and *Bambiraptor* is oriented laterally, as in *Bambiraptor* AMNH FR 30554 (Burnham, 2004), *Sinornithosaurus* IVPP V12811 (Xu, Wang & Wu, 1999), *Microraptor* IVPP V13353 and LHV 0026 (Xu et al., 2003; Gong et al., 2012), and *Changyuraptor* HG B016 (Han et al., 2014), which more closely approaches the condition in birds than that of velociraptorine dromaeosaurids and most troodontids, whose glenoid fossa oriented to caudoventrally as mentioned previously (Table S1 and Fig. 4).

The acromion process of the scapula of these potentially volant dromaeosaurids is well-developed, with its apex projected from the scapular equal or longer than the scapular glenoid, and is elongated cranially, extending over the articular surface to the coracoid, as clearly preserved in *Bambiraptor* AMNH FR 30554, *Sinornithosaurus* IVPP V12811, *Rahonavis* UA 8656 and *Unenlagia* MCF PVPH 78 (Fig. 4). In volant dromaeosaurids, the coracoid is mediolaterally narrow forming a distinct neck below the glenoid fossa and scapula articular surface that separates the omal articular surface from the coracoid body, as observed in *Bambiraptor* AMNH FR 30554, *Wulong* D2933 and *Sinornithosaurus* IVPP V12811 (Fig. 4) (Burnham, 2004). This neck of the coracoid of *Bambiraptor* was considered to be caused by loss of the medial margin of the coracoid which also occurs in *Buitreraptor* MPCN-PV-598 and MPCA 245 (Gianechini et al., 2018; Novas et al., 2018). However, in previous description the author stated that the left coracoid of *Bambiraptor* is complete (Burnham, 2004), and similar morphology is also observed in microraptorines. Then the neck of the coracoid is probably not a preservation artifact.

The acrocoracoid process is well-developed and located close to the glenoid fossa, as preserved in *Bambiraptor* MNH FR 30554 (Burnham, 2004), *Zhongjianosaurus* IVPP V22775 (Xu, Qin & Palasiatica, 2017), *Tianyuraptor* STM1-3 (Zheng et al., 2010), *Wulong* D2933 (Poust et al., 2020), and *Sinornithosaurus* IVPP V12811 (Xu, Wang & Wu, 1999). When the coracoid foramen is present, the acrocoracoid process is located between the foramen and the glenoid fossa, as in *Sinornithosaurus* IVPP V12811 (Xu, Wang & Wu, 1999).

### Anchiornithidae

Anchiornithidae includes the most basal known clade of avialan, and includes *Anchiornis*, *Eosinopteryx*, *Xiaotingia* and *Serikornis* (Pol & Goloboff, 2020). The phylogenetic placement of the Anchiornithidae has been contentious. It has been placed within Troodontidae (Xu et al., 2011; Lee & Worthy, 2012; Brusatte et al., 2014; Gianechini, Ercoli & Díaz-Martínez, 2020), or resolved as an early diverging lineage of avialans (Agnolín & Novas, 2013; Agnolín et al., 2019; Pittman & Xu, 2020; Novas et al., 2021b). One recent study found that anchiornithids have potential for partially powered flight similar to that inferred for *Microraptor, Rahonavis, Jeholornis*, and *Confuciusornis* (Pei et al., 2020).

**Figure 5:**
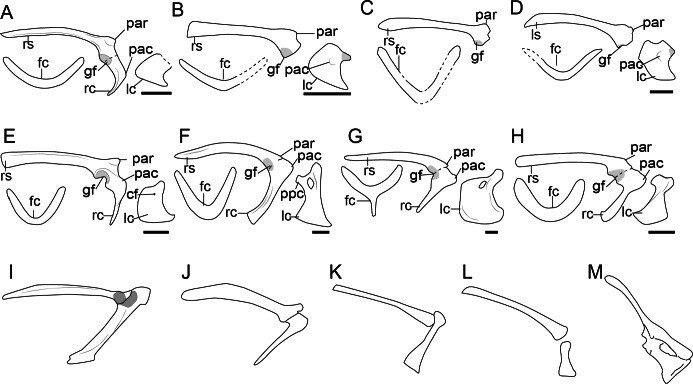
Comparison of the pectoral girdle of Anchiornithidae and basal birds, as well as pectoral girdle morphology change (lateral view) of flightless terrestrial bird. (A) *Anchiornis* right scapulocoracoid in lateral view (showing the medial and lateral crash L-shape), furcula in cranial view based on LPM-B00169 and left coracoid in cranial view (showing the subaquatic shape) based on BMNHC PH804 (originally right side) modified after Hu et al. (2009), Pei et al. (2017), and IVPP V14378 based on personal observation; (B) *Eosinopteryx* based on YFGP-T5197 modified after Godefroit et al. (2013); (C) *Xiaotingia* based on STM 27-2 modified after Xu et al. (2011) (originally no scale bar); (D) *Serikornis* based on PMOL-AB00200 modified after Lefèvre et al. (2017). (E) *Archaeopteryx* right scapulocoracoid based on Mexberg specimen modified after Wellnhofer (2009), and left coracoid and furcula based on 10th specimen modified after Mayr, Pohl & Peters (2005); (F) *Jeholornis* right scapulocoracoid based on STM after O’Connor et al. (2018), left coracoid based on STM 2-49 and IVPP V13886 modified after Wang et al. (2022a), Wang et al. (2022b), furcula based on YFGP-yb2 modified after Lefèvre et al. (2014); (G) *Sapeornis* right scapulocoracoid based on IVPP V12698, furcula and left coracoid based on IVPP V13276 modified after Zhou & Zhang (2003a), Zhou & Zhang (2003b); (H) *Confuciusornis* right scapulocoracoid based on IVPP V13168 and GMV-2132 modified from Li (2010), Chiappe et al. (1999); furcula based on GMV-2131 modified after (Chiappe et al., 1999), left coracoid based on IVPP V16066 modified after Li (2010). (I) crown birds based on *Gallus*; (J) *Patagopteryx* based on MACN-N-11 modified after Chiappe (2002); *Rallus* subadult (47 days) (K) and juvenile (17 days) (L) modified from Olson (1973); (M) *Struthio* modified from McGowan (1982). In (B–D), scapula in lateral view, coracoid and furcula in cranial view. In (E–H), Right scapulocoracoid in lateral view, left coracoid and furcula in ventral view. Reconstructed portion marked with dash line; gray color indicates the articular surface for humerus when identical from the specimen during reconstruction. Abbreviations: ppc, procoracoid process; Abbreviations: cf, coracoid foramen; fc, furcula; gf, glenoid fossa; lc, left coracoid, ls, left scapula; rc, right coracoid; rs, right scapula; pac, acrocoracoid process; par, acromion process; ppc, procoracoid process. In (A–H), Scale bar = 1 cm. In (I–M), not to scale.

Hundreds of specimens of *Anchiornis* have been reported (Xu et al., 2009; Hu et al., 2009; Pei et al., 2017; Guo, Xu & Jia, 2018). Among them, the scapula and coracoid of the relative smaller specimen BMNHC PH 804 (humerus length 44.5–45.7 mm) are connected but unfused (Pei et al., 2017), while a larger specimen LPM-B00169 (humerus length 69.0 mm) is described as having a fused scapulocoracoid (Hu et al., 2009). In other anchiornithids known from single specimens the two elements are separate (Figs. 5A–5D), *e.g.*, in *Eosinopteryx* YFGP-T5197, *Xiaotingia* STM 27-2, and *Serikornis* PMOL-AB00200 (Xu et al., 2011; Godefroit et al., 2013; Lefèvre et al., 2017). Claims that the holotypes of *Eosinopteryx* and *Xiaotingia* are based on adult material are equivocal (Xu et al., 2011; Godefroit et al., 2013), and the only known specimen of *Serikornis* is considered by some to be a subadult (Lefèvre et al., 2017). Therefore, we suggest that the synchondrosis between the scapula and coracoid in anchiornithids fuses late in ontogeny, as in oviraptorosaurs and other non-avialan paravians.

Anchiornithids have a smaller articular surface between the scapula and coracoid than observed in oviraptorosaurs and troodontids (as in *Xiaotingia* STM 27-2 and *Serikornis* PMOL-AB00200), a laterally oriented glenoid fossa as in *Anchiorni* s IVPP V14378 (Fig. 5A), an approximate right angle formed by the scapula and coracoid in lateral view (Fig. 5A) (Hu et al., 2009). In the lateral view, the fused scapulocoracoid of *Anchiornis* LPM-B00169 is “L-shape”, indicating the cranial orientation of the coracoid body (Fig. 5A). However, in contrast to *Microraptor*, the acromion of anchiornithids is not elongated cranially over the coracoid articular surface (Figs. 5A–5D), resembling the condition in oviraptorosaurs (Fig. 2). The coracoid of *Anchiornis* BMNHC PH804 and *Eosinopteryx* YFGP-T5197 is trapezoidal in cranial view with expanded sternal ends and concave lateral margin (Xu et al., 2009; Hu et al., 2009; Godefroit et al., 2013), resembling the plesiomorphic pennaraptoran condition. While in *Serikornis* PMOL-AB00200, the concave medial margin of the coracoid resembling that of the volant dromaeosaurids is probably a preservational artifact since the coracoid is overlapped by the furcula (Lefèvre et al., 2017).

Thus, it is not strictly correct to say that condition in anchiornithids is closer to birds than that of dromaeosaurids like *Microraptor.* Some characterstics, but not all, are consistent with birds whereas others resemble more basal pennaraptorans. This is consistent with the limited powered flight capacity inferred from their feather structure, forelimb proportions, and homoplasy related to the repeated evolution of flight in pennaraptorans (Pei et al., 2020), and suggests acquisition of some flight adaptive skeletal features in volant dromaeosaurids or the common ancestor of dromaeosaurids independent and in parallel to avialans.

### Basal birds (non-ornithothoracine avialans)

Basal birds here representative *Archaeopteryx* and later-diverging non-ornithothoracine avialans. Besides *Archaeopteryx* (here regards as the most basal bird), basal birds include other long tail birds such as *Jeholornis*, basal pygostylians *Sapeornis,* as well as confuciusornithids and jinguofortisids, clades that are interpreted as crownward of *Jeholornis* and *Sapeornis* (Wang, Stidham & Zhou, 2018; Wang, O’Connor & Zhou, 2019; Wu et al., 2021b).

The scapula of basal birds are located at the dorsal side of the thoracic cage and parallel to the vertebral column, similar to the position of extant birds (Fig. S1), as shown in the Munich specimen of *Archaeopteryx* (Wellnhofer, 2009; Wu et al., 2021a), *Jeholornis* IVPP V 13353 (Zhou & Zhang, 2003a), *Sapeornis* IVPP V 13396 (Provini, Zhou & Zhang, 2009), and confuciusornithids IVPP V 11977 (Zhang, Zhou & Benton, 2008) and DNHM D2454 (Zhang et al., 2009).

All birds possess a localized articular surface between the scapula and coracoid (Figs. 5E–5H), *e.g.*, *Archaeopteryx* 10th (Thermopolis) specimen (WDC-CSG-100) (Ostrom, 1976; Elzanowski, 2001; Chiappe & Witmer, 2002; Rauhut, Foth & Tischlinger, 2018), *Jeholorni* s IVPP V13274 and V13886 (Zhou & Zhang, 2002; Wang et al., 2022b), *Sapeornis* IVPP V13276 (Zhou & Zhang, 2003b; Wang et al., 2022b), and *Confuciusornis* IVPP V16066 (Fig. 5H) (Li, 2010), similar to that of *Buitreraptor* and *Bambiraptor* while contrast to the long articular surface of troodontids, but the joint type between the scapula and coracoid is unclear.

Although these two elements were thought to be separate in most basal birds, like *Archaeopteryx*, *Jeholornis* and *Sapeornis* (Zhou & Zhang, 2003b; Mayr, Pohl & Peters, 2005; Wang, Stidham & Zhou, 2018), fusion of the scapulocoracoid was also reported in the Maxberg, Solnhofen, and Munich specimens of *Archaeopteryx* (Wellnhofer, 2009), as well as in the Confuciusornithidae and Jinguofortisidae (Wang, Stidham & Zhou, 2018; Wang, O’Connor & Zhou, 2019; Wu et al., 2021b). Hence, the scapula and coracoid may evolve to be unfused in basal birds crownward of *Archaeopteryx*, with that in the Confuciusornithidae and Jinguofortisidae being secondarily fused (Fig. 1) (Wu et al., 2021a). Alternatively, separation between the scapula and coracoid evolved independently in *Jeholornis*, *Sapeornis* and Ornithothoraces or in a clade formed by *Sapeornis* and Ornithothoraces (the sister taxon to Ornithothoraces is alternatively resolved as either *Sapeornis* or the Confuciusornithidae in various published phylogenetic analyses) (Gianechini et al., 2018; Wang, Stidham & Zhou, 2018; Agnolín et al., 2019; Wang et al., 2022a)–both interpretations are equally parsimonious.

**Figure 6:**
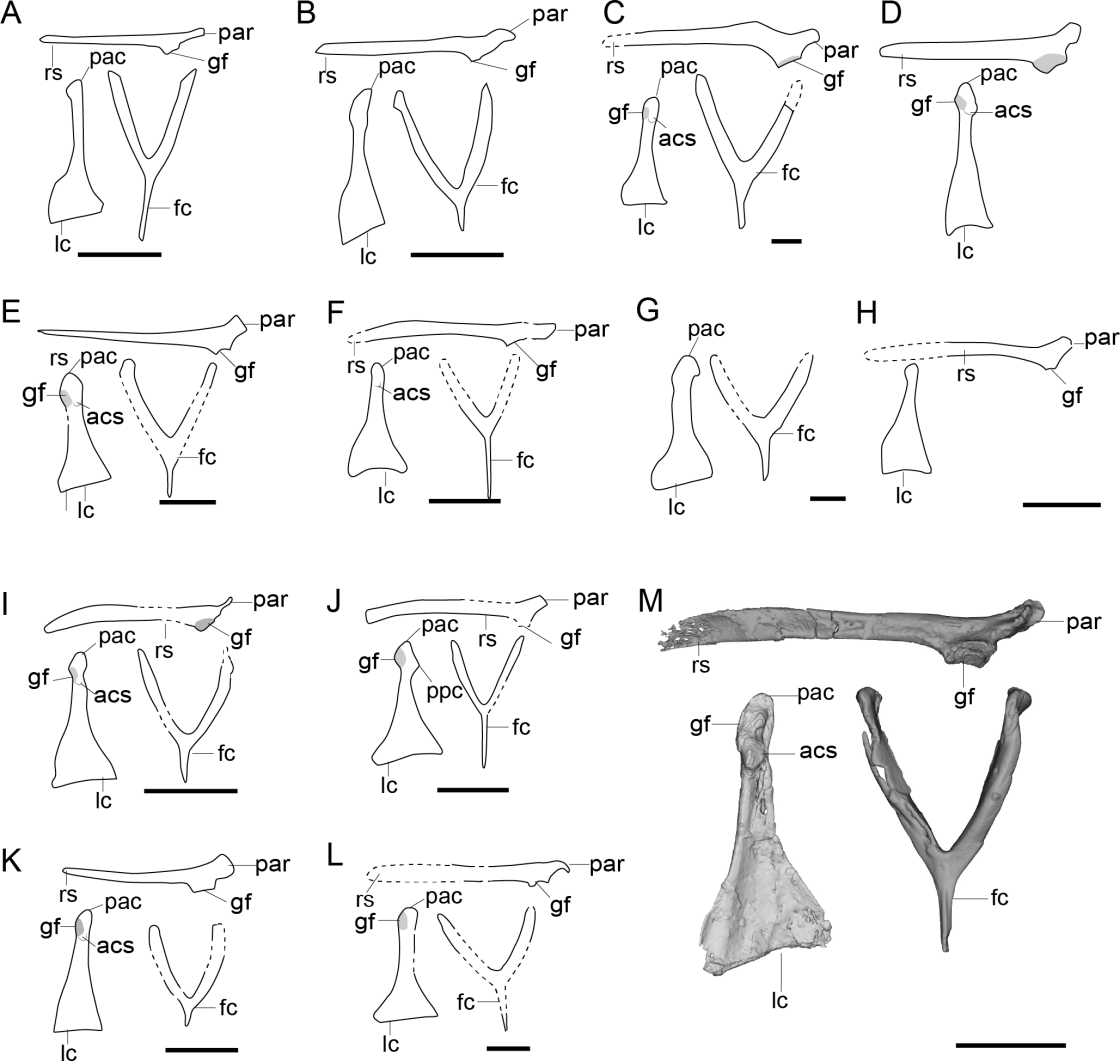
Comparison of the pectoral girdle of Enantiornithes. (A) *Eoalulavis* based on MCCM-LH-13500 modified after Sanz et al. (1996); (B) *Concornis* based on MCCM-LH-1184 (Serrano et al., 2018); (C) *Elsornis* based on MPD-b 100/201 (Chiappe et al., 2007); (D) *Enantiornis* based on PVL-4035 (coracoid) and PVL-4055 (scapula) (Chiappe & Witmer, 2002); (E) *Bohaiornis* based on IVPP V17963 modified after Li et al. (2014); (F) *Dunhuangia* based on GSGM-05-CM-030 after Wang et al. (2015a); (G) *Xiangornis* based on PMOL-AB00245 after Hu et al. (2012); (H) *Eocathayornis* based on IVPP V10916 after Zhou (2002); (I) *Junornis* based on BMNHC PH 919 (Liu et al., 2017); (J) *Protopteryx* based on BMNHC Ph 1158 after Chiappe et al., 2019a; Chiappe et al., 2019b; (K) *Shangyang* based on IVPP V25033 after Wang & Zhou (2019); (L) *Parapengornis* based on IVPP V18687 after (Hu, O’Connor & Zhou, 2015); (M) *Piscivorenantiornis* based on IVPP V22582 (Wang et al., 2022a; Wang et al., 2022b). Reconstructed portion marked with dash line; gray color indicates the articular surface for humerus when identical from the specimen during reconstruction. Scapula and coracoid in dorsal view, furcula in ventral view. Abbreviations: acs; articular surface for the scapula, cf, coracoid foramen; fc, furcula; gf, glenoid fossa; lc, left coracoid, ls, left scapula; rc, right coracoid; rs, right scapula; pac, acrocoracoid process; par, acromion process; ppc, procoracoid process. Scale bar = 1 cm.

**Figure 7:**
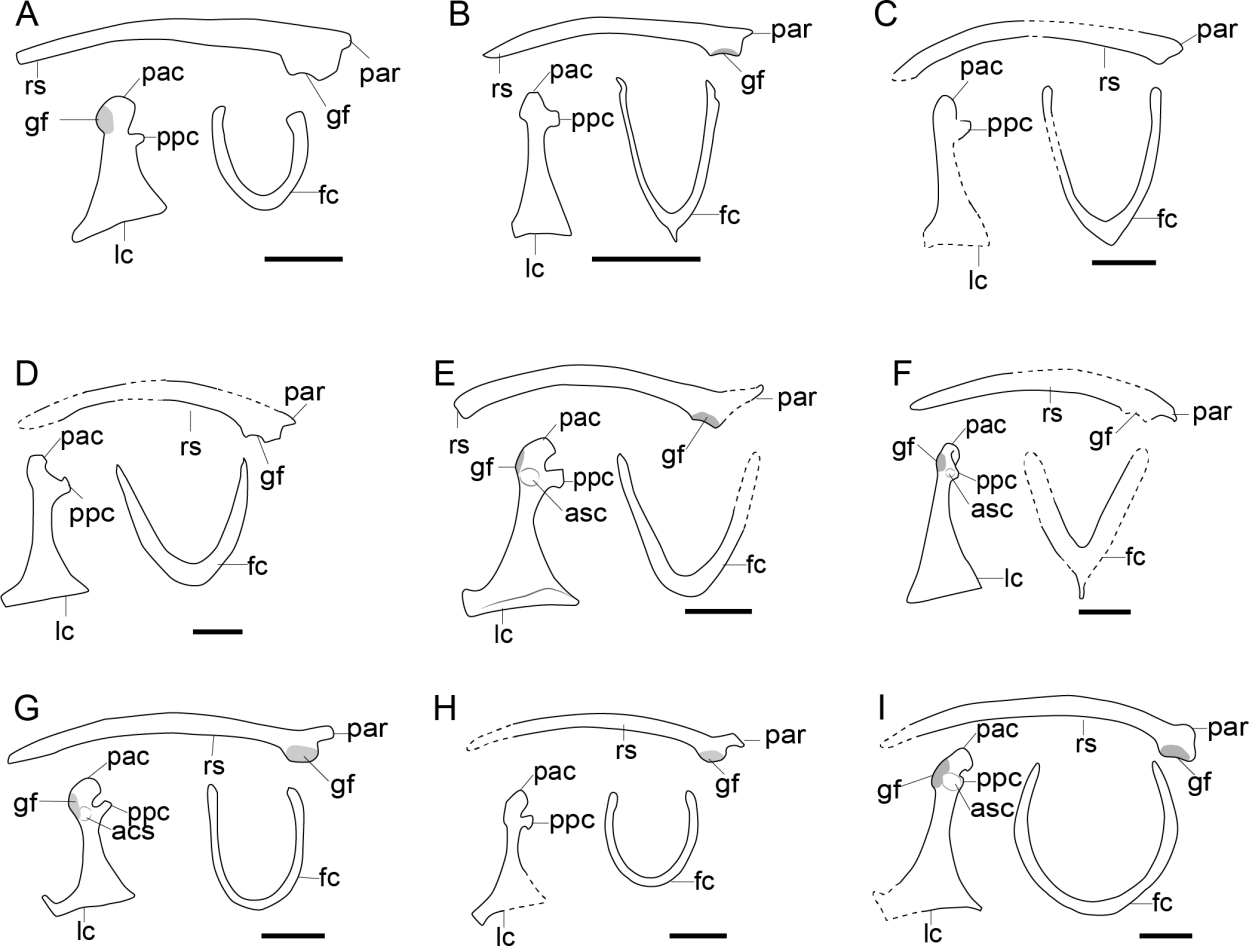
Comparison of pectoral girdle of Ornithuromorpha. (A) *Archaeorhynnchus* based on IVPP V17091 and IVPP V14287 modified from Zhou, Zhou & O’Connor (2013); (B) *Hongshanornis* based on IVPP V14533 (Zhou & Zhang, 2005); (C) *Archaeornithura* based on STM7-145 modified from Wang et al. (2015b); (D) *Abitusavis* (Yanornithidae), based on IVPP V14606 modified from Wang et al. (2020a); (E) *Yixianornis* based on IVPP V12631 (Zhou & Zhang, 2001); (F) *Mengciusornis* based on IVPP V26275 modified from Wang et al. (2020b); (G) *Gansus* based on CAGS -IG-04-CM-003 and GSGM-07-CM-006 (You et al., 2006; Wang et al., 2016) and 05-CM-026; (H) *Ambiortus* based on PIN 3790/271 modified from O’Connor & Zelenkov (2013); (I) *Ichthyornis* based on SMM 2503, YPM 1755, YPM 1733, YPM 1773 (Clarke, 2004). Reconstructed portion marked with dash line; gray color indicates the articular surface for humerus when identical from the specimen during reconstruction. Scapula and coracoid in dorsal view, furcula in ventral view. Abbreviations cf, coracoid foramen; fc, furcula; gf, glenoid fossa; lc, left coracoid, ls, left scapula; rc, right coracoid; rs, right scapula; pac, acrocoracoid process; par, acromion process; ppc, procoracoid process. . Scale bar 1 cm. B, E, G, I are drawn by the author.

However, a tightly connected scapula and coracoid forming an L-shape in lateral view are present in all non-ornithothoracines basal birds (e.g., *Jeholornis* YGFP-yb2 (Lefèvre et al., 2014), *Sapeornis* IVPP V12698 (Zhou & Zhang, 2003b)), resembling the preservation in some troodontids, dromaeosaurids, and anchiornithids (Figs. 2–5). This is not observed in the ornithothoracines (Figs. 6, 7). Considering that precise ontogenetic age is unverified through histology in most specimens, the absence of fusion between these two elements in basal birds may also be due to somatic immaturity in most specimens, as in non-avialan pennaraptorans, and the separate, unfused scapula and coracoid may have only evolved in ornithothoracines. However, even if the scapula and coracoid of basal birds crownward of *Archaeopteryx* do not become fused with somatic maturity as in other early pennaraptorans, the joint between these two elements is still clearly different from that of ornithothoracines, in which the two elements are clearly preserved free (and not forming the “L-shape” in lateral view). In contrast to other basal birds, the scapula and coracoid of *Confuciusornis* become fused early in ontogeny well before skeletal maturity, forming a synostosis. This is considered an autapomorphy of this lineage (Wang, O’Connor & Zhou, 2019; Wu et al., 2021a).

The angle between the scapula and coracoid of basal birds is less than 90°, except for *Archaeopteryx*, in which the angle is approximately 90° (Fig. 5E). Correspondingly, the position of the scapulocoracoid inflection is within the body of coracoid in *Archaeopteryx* and basal-ward pennaraptorans (*e.g.*, *Anchiornis* and *Buitreraptor*), but between the scapular and coracoid in Ornithothoraces (Novas et al., 2021b). The lateral orientation of the glenoid fossa in *Sapeornis* (Wang et al., 2022b), *Archaeopteryx* (Rauhut, Foth & Tischlinger, 2018), and *Confuciusornis* (Martin et al., 1998; Li, 2010) are similar to the condition in microraptorines, unenlagiines and anchiornithids (Figs. 4, 5A—5D). The glenoid fossa of *Jeholornis* IVPP V13353 and YFGP-yb2 was described to be dorsolaterally oriented (Zhou & Zhang, 2003a; Lefèvre et al., 2014), but in YFGP-yb2 the glenoid is not as dorsally positioned as that of extant bird. Deformation is a common phenomenon in fossil preservation. Based on the preservation status of majority specimens, we tend to believe that the glenoid fossa of *Jeholornis* is laterally oriented, as in other basal birds (Figs. 5E, 5G, 5H).

The scapular acromion process in basal birds is elongate and extends cranially over the coracoid articular surface (Figs. 5E–5H) (Chiappe et al., 1999; Zhou & Zhang, 2003a; Zhou & Zhang, 2003b; Wellnhofer, 2009; Lefèvre et al., 2014), to articulate with the epicleideal process of furcula along its cranial tip as in ornithothoracines (Fig. 1) (Wang et al., 2022b). The acromion of *Archaeopteryx* is dorsally deflected slightly (Fig. 5E), while that of the *Jeholornis* and *Sapeornis* extends cranially (Figs. 5F, 5G) (Zhou & Zhang, 2003b; Wang et al., 2022b). The acromion of *Confuciusornis* appears to be less developed, developed only as a small bump in lateral view (Fig. 1D). The epicleideal process of the furcula was proposed to articulate with a small process on the medial side of the scapulocoracoid of *Confuciusornis* (Wu et al., 2021b). These two features together with the fused scapulocoracoid, make *Confuciusornis* a morphological outlier among basal birds.

Reconstructions based on 3D CT data demonstrate that the scapuloclavicular joint moved from the dorsal side of the coracoscapular joint in non-avialan pennaraptorans, to the medial side of the coracoscapular joint in ornithothoracines, and some basal birds like *Sapeornis* (Figs. 1, 5G) (Wang et al., 2022b). Under the latter circumstances, the acromion of the scapula is not visible when the pectoral girdle is in lateral view (Fig. 1) (Wang et al., 2022b). Hence, the acromion process of *Confuciusornis* may extend cranially along the medial side of the pectoral girdle like more derived ornithothoracines, and the small medial side process in articulation with the furcula found by Wu et al. (2021b) may in fact be the cranial end of the acromion process, and the joint between is the scapuloclavicular joint rather than the coracoclavicular joint.

In *Jeholornis*, the acromion process is visible in dorsally preserved scapula of specimens like IVPP V13353 and YFGP-yb2 (Zhou & Zhang, 2003a; Lefèvre et al., 2014), but not in lateral view on either side of the fused scapulocoracoids of STM2-19 (O’Connor et al., 2018). Additionally, given the similarities between the shoulder girdle of *Jeholornis* and more crownward birds, like the procoracoid process and acrocoracoid process, we believe that the scapuloclavicular joint is more likely to be medial than the coracoscapular joint, as in the *Confuciusornis* and *Sapeornis*. In contrast, in some other basal birds as *Archaeopteryx*, the cranial tip of the acromion (to which the furcula articulates) is clear visible in lateral view of the fused scapulocoracoid in lateral view (Fig. 5E) (Wellnhofer, 2009; Lefèvre et al., 2014), indicating the scapuloclavicular joint is still located dorsal than the coracoscapular joint.

The morphology of the coracoid in non-ornithothoracine birds varies greatly. *Archaeopteryx* has a plesiomorphically trapezoid coracoid with a shortened omal end, convex medial margin and concave lateral margin, and a acrocoracoid process that is located more dorsal than the supracoracoid foramen and almost level with the glenoid fossa (Fig. 5E) (Ostrom, 1976; Mayr, Pohl & Peters, 2005). The coracoid of *Sapeornis* is similar to that of the *Archaeopteryx* in shape, but the acrocoracoid process is more dorsocranially elongated, and located at the same level as the glenoid fossa (Fig. 5G) (Zhou & Zhang, 2003a; Zhou & Zhang, 2003b; Wang et al., 2022b), as is the acrocoracoid process of *Jeholornis* and *Confuciusornis* (Fig. 5F, H) (Zhang et al., 2009; Li, 2010; Lefèvre et al., 2014), which is more dorsally located and prominent compared to non-avialan paravians (Figs. 3–5).

Although elongated relative to more basal taxa and thus described as “strut-like”, the morphology of the coracoid in *Jeholornis* and *Confuciusornis* show some differences to that of extant birds, having a relative thicker coracoid ‘neck’ (Figs. 5F, 5H). This fairly robust coracoid neck is also observed in the stem-most ornithuromorph *Archaeorhynchus* IVPP V17091 (Zhou, Zhou & O’Connor, 2013). The coracoid of *Jeholornis* is unique relative to that in other basal birds with a well-developed procoracoid process (Fig. 5F) (Turner, Makovicky & Norell, 2012; Lefèvre et al., 2014), being the stem-most appearance of this feature but represents a local apomorphy. The procoracoid process in *Jeholornis* is located on the omal portion of the medial margin of the coracoid, directed dorsomedially and at the same level as the large supracoracoid foramen (Turner, Makovicky & Norell, 2012; Lefèvre et al., 2014; Wang et al., 2022b). The procoracoid process may participate in the coracoscapular joint of *Jeholornis*, as in some extant birds (Baumel et al., 1993; Wang et al., 2022b).

### Non-neornithine Ornithothoraces

Ornithothoraces comprises the sister groups: Enantiornithes and Ornithuromorpha, and the latter includes crown birds (neornithes) (Livezey & Zusi, 2007; Wang, Stidham & Zhou, 2018). The pectoral girdle of non-neornithine ornithothoracines displays the essential morphological characteristics of extant birds, including an unfused scapula and coracoid, strut-like and ventrally oriented coracoid body, coracoscapular joint located medial and not dorsal to the glenoid fossa, glenoid fossa located below the pronounced acrocoracoid process and oriented dorsolaterally, well-developed acromion process of the scapula elongated cranially over the coracoid articular surface, and articular surface with the furcula on the cranial tip of the scapular acromion (Chiappe & Witmer, 2002; Zhou, 2013). Although the disarticulation of the scapula and coracoid prevent accurate measurement of the scapula-coracoid angle, the morphology of the articular surfaces and CT reconstructions support an acute angle as in extant birds in both enantiornithines and ornithuromorphs (Mayr, 2017; Wang et al., 2022b).

Except for the presence of an acrocoracoid dorsal to the glenoid fossa, and dorsolaterally oriented glenoid fossa, other derived characters were already in place in basal birds. Among the three main joints forming the triosseal canal in extant birds, two have formed a similar state in non-neornithine ornithothoracines, even in some basal birds, namely the scapuloclavicular joint between the omal tip of the epicleideal process of the furcula and the cranial tip of the acromion process of the scapula, as well as the unfused and localized coracoscapular joint between the scapula and coracoid (see the previous analysis). This indicates that the joint between the epicleideal process of the furcula and the acrocoracoid process of the coracoid is the last evolutionary step in the formation of an osseous triosseal canal. Besides these similarities, there are significant differences between the pectoral girdle of these two clades discussed separately below.

### Enantiornithes

The scapular acromion process of the scapula in enantiornithines is well-developed and elongated cranially, extending over the articular surface to the coracoid (Fig. 6). In most enantiornithines, the acromion is bend medially, except for *Concornis* and Pengornithidae, which have a hocked acromion (Figs. 6B,6L) (Zhou, Clarke & Zhang, 2008; Hu, Zhou & O’Connor, 2014; Wang et al., 2014; Hu, O’Connor & Zhou, 2015; Serrano et al., 2018). Most enantiornithines, with the exception of some of the Pengornithidae, have a straight acrocoracoid process that extends dorsally. The acrocoracoid is straight in *Eopengornis* STM 24-1 (Wang et al., 2014), slightly medially inclined in Pengornithid indet. IVPP V18632 (Hu, Zhou & O’Connor, 2014), but very weekly developed in *Parapengornis* IVPP V18687 (Hu, O’Connor & Zhou, 2015). The procoracoid process on the coracoid is absent with the exception of the basal *Protopteryx* IVPP V11844 and BMNHC Ph 1158 (Fig. 6), which has a small, triangular, procoracoid-like medial process (Zhang & Zhou, 2000; Chiappe et al., 2019a).

The scapular articular surface of the coracoid is weakly convex and the coracoid articular surface of the scapula is concave, which is opposite the condition in extant birds and considered to be an autapomorphy and diagnostic feature of enantiornithines. However, recently discoveries may challenge this view. In *Alethoalaornis* LPM00009 the coracoid articular surface on the scapula is reportedly slightly convex (Li et al., 2007) but this cannot be confirmed in the provided figures and preservation in these specimens is poor making support for this interpretation unlikely in this taxon. In the basal pengornithids *Parapengornis* IVPP V18687 and Pengornithid indet. V18632, the scapular articular surface on the coracoid is shallowly concave suggesting this feature is not diagnostic of the entire clade or that the condition in pengornithids is an autapomorphy of this clade (Wang et al., 2022b). The phylogenetic position of the Pengornithidae is controversial (Zelenkov, 2017), and requires further study.

3D reconstructions based on CT data show that the distance between the acrocoracoid process and the epicleideal process in enantiornithines is relatively far, indicating that both an articulation between these two elements like that in extant birds and a bony, closed triosseal canal are absent in this clade (Wang et al., 2022b).

### Non-neornithine Ornithuromorpha

In contrast to enantiornithines, the articular surface between the scapula and coracoid in non-neornithine ornithuromorphs is convex on the scapula and concave on the coracoid, as in *Yixianornis* IVPP V12631, *Mengciusornis* IVPP V26275, *Gansus* CAGS-IG-04-CM-003, and *Ichthyornis* YPM 1733 (Fig. 7) (Zhou & Zhang, 2001; Clarke, 2002; You et al., 2006; Wang et al., 2020b). This morphology is often described as a “ball and socket” joint (Turner, Makovicky & Norell, 2012), but this interpretation is debated (Mayr, 2021). Furthermore, a flat articular surface for the scapula on the coracoid has evolved in crown birds at least 13 times (Mayr, 2021).

Except for *Apsaravis* IGM 100/1017 (Clarke & Norell, 2002), *Patagopteryx* MACN-N-11 (Chiappe, 1996; Chiappe & Witmer, 2002), and *Baptornis* KUVP 2290 (Bell & Chiappe, 2020), all known Mesozoic ornithuromorphs possess a procoracoid process on the coracoid, including the Late Cretaceous flightless *Hesperornis* (Fig. 7). Instead of a well-developed procoracoid process, *Apsaravis* IGM 100/1017 possess a very slight bulging on the medial surface of the coracoid (Clarke & Norell, 2002), which may actually be the residual of a secondarily reduced or taphonomically lost procoracoid process. The procoracoid process has been lost in flightless *Patagopteryx* (Chiappe, 1996; Chiappe & Witmer, 2002), *Baptornis* KUVP 2290 (Bell & Chiappe, 2020), and many neornithines as well, such as the volant *Pavo muticus* (Wang et al., 2022b), and flightless *Thambetochen chauliodus* (Feduccia, 1981). However, in the coracoid of some flightless taxa such as the Mesozoic *Pasquiaornis* RSM P1988.9 (Bell & Chiappe, 2020) and extant *Struthio camelus* (Vickaryous & Hall, 2006) the procoracoid process is retained, hence the relationship between the loss of the procoracoid process and flight in birds is unclear.

The acrocoracoid process in volant ornithuromorphs is well-developed (Fig. 7) and more robust than that of enantiornithines (Fig. 6). This process varies from straight (as in *Archaeorhynchus* IVPP V17091 (Zhou, Zhou & O’Connor, 2013), *Hongshanornis* IVPP 14533 (Zhou & Zhang, 2005), and *Yanornis* IVPP V12558 (Zhou & Zhang, 2001), Fig. 7), to medially inflected (as in *Yixianornis* IVPP V12631 (Zhou & Zhang, 2001), *Gansus* CAGS-IG-04 CM-004 (You et al., 2006), and *Mengciusornis* IVPP V26275 (Wang et al., 2020b)), to hooked in some members of the derived, Late Cretaceous clade Ornithurae (as in *Ichthyornis* YPM 1733 (Clarke, 2004)). The ‘neck’ of the coracoid of the basal ornithuromorph *Archaeorhynchu* s IVPP V17091 is broader than that of other ornithuromorphs (Fig. 7) (Zhou & Zhang, 2006; Zhou, 2013), but whether the acrocoracoid process has an articular surface for the furcula cannot be determined from the preservation of currently available specimens. In at least one Early Cretaceous ornithuromorphs *Yixianornis* IVPP V13631, the articular surface is clearly visible on the medial surface of the acrocoracoid process (Clarke, Zhou & Zhang, 2006), resembling the articular surface in extant birds (Baumel et al., 1993) and indicating the presence of a modern triosseal canal in *Yixianornis*.

### Secondarily flightless birds

There are several Mesozoic birds that are interpreted as secondarily flightless, *e.g.*, *Patagopteryx, Elsornis,* and *Hesperornis* (Chiappe & Witmer, 2002; Chiappe et al., 2007). Flightlessness has also evolved many times in different lineages of crown birds, such as *Thambetochen chauliodus* (Anatidae) (Olson & Wetmore, 1976), *Strigops habroptilus* (Psittaciformes) (Livezey, 1992), several lineages of paleognaths (Houde, 1986), species of Phorusrhacidae (Cariamiformes) (Alvarenga & Höfling, 2003), and many island rails (Rallidae) (Olson, 1973).

Among these birds whose flight capability is secondarily reduced, the pectoral girdle shows some important morphological changes including (Figs. 5I–5M): fusion of the scapula and coracoid in the flightless paleognaths, as in the ostrich (McGowan, 1982); decrease in the relative proportions of the forelimb and pectoral girdle relative to entire skeleton, as in flightless paleognaths and flightless rails (Olson, 1973); increase in the scapulocoracoid angle to 90°, as in flightless rails and *Strigops* (Olson, 1973; Livezey, 1992), or greater, as in flightless paleognaths (McGowan, 1982); reduction of the scapular acromion and acrocoracoid process of the coracoid, resulting in the glenoid located at the proximal end of coracoid rather than below it, as in flightless paleognaths and *Patagopteryx* (McGowan, 1982; Chiappe & Witmer, 2002); increase in the space between the left and right sternal articulation of the coracoids, as in *Patagopteryx*, *Strigops* and flightless paleognaths (McGowan, 1982; Livezey, 1992; Chiappe & Witmer, 2002); degeneration of the furcula, into two separate clavicles as in *Strigops* (Livezey, 1992), reduced and unfused clavicles in the ostrich and even the complete loss of the furcula in the kiwi (McGowan, 1982); as well as the loss of the triosseal canal as in the flightless paleognaths (McGowan, 1982). These characters represent the complete spectrum of pectoral girdle degeneration observed in different flightless lineages, though all these features do not simultaneously co-occur in any single species. Among these pectoral girdle transformations, th**e** fusion of the scapula and coracoid**,** obtuse scapulocoracoid angle, degeneration of the scapular acromion and acrocoracoid processes, and loss of the osseous triosseal canal resemble the plesiomorphic conditions variably observed in non-avialan pennaraptorans (Fig. 2).

## Morphology and Function Transformations of Pennaraptoran Pectoral Girdle Associated with Flight Evolution

The pectoral girdle morphology changed dramatically during pennaraptoran evolution from oviraptorosaurs to birds, including (but not limited to) the location and morphology of the coracoscapular joint and the scapuloclavicular joint, the location and orientation of the glenoid fossa, the morphology and orientation of the coracoid, procoracoid and acrocoracoid process of the coracoid, and acromion process of the scapula (Fig. 1, Table 1). These can be divided into three main transformations.

First, the change in the orientation of the glenoid fossa, from the caudoventral orientation in oviraptorosaurs (Osmólska, Currie & Barsbold, 2004), to relatively lateral orientation in basal dromaeosaurids, anchiornithids and basal birds (Pittman & Xu, 2020; Wang et al., 2022b), culminating in the dorsolateral orientation present in ornithothoracines (Fig. 1) (Baumel et al., 1993). This transformation allowed the forelimb of ornithothoracines greater range of motion and to be fully elevated dorsally above the vertebral column to accommodate the extensive wing stroke utilized in the powered, flapping flight of modern birds (Novas et al., 2021b).

In extant birds, *Rhea* with laterally oriented glenoid fossa and sub-ventrally oriented glenoid main axis can certainly extend the forelimb laterally, as, but may not be able to perform effective flapping wing-strokes, being limited in their dorsal amplitude (Novas et al., 2020). The humeral articular surface on the scapula (scapular half of the glenoid) in the basal bird *Sapeornis* is concave and possesses a raised lip along the dorsal to caudal margin (Wang et al., 2022b), which would have been further accentuated *in vivo* by cartilage tissue. Thus dorsal movement of the wing of the *Sapeornis* would likely have been prevented, limiting the vertical range of its wing-stroke and thus the amount of lift generated by its flapping motion. This is somewhat consistent with interpretations that *Sapeornis* would have relied primarily on soaring flight (Bell & Chiappe, 2011; Serrano & Chiappe, 2017; Serrano et al., 2020). However, soaring birds must be able to flap to achieve soaring altitude, which may suggest that *Sapeornis* instead utilized gliding flight. A reduction in wing loading can offset this limitation to some extent, but wing loading in basal birds is not considered to be significantly lower than that of ornithothoracines (Pei et al., 2020). This strongly suggests limits in the powered flight ability present in some basal birds, specifically the ability for ground take-off and sustained long distance flight, which require greater lift than passive gliding flight. This is also consistent with the presence of claws on the hands of basal birds that can be used for climbing and resemble those present in juvenile hoatzins (Abourachid et al., 2019).

Thus, extensive flapping motion of the forelimb similar to extant birds probably did not evolve until the appearance of a laterodorsally oriented glenoid fossa in ornithothoracines. This coincides with the appearance of a sternal keel, which provided an expanded surface for the attachment of the *M. pectoralis major*, the main muscle responsible for the down-stroke of the wing beat. The appearance of both a glenoid that permits the full range of flapping movement and increased surface area for the muscle that powers it in ornithothoracines may suggest fully equipped powered flight is limited to this clade. Further increases in the size of the sternal keel in ornithuromorphs relative to enantiornithines indicate the ability to generate greater power and lift through the wing stroke. An enlarged sternal keel likely evolved in parallel in enantiornithines by the Late Cretaceous, as evidenced by the well-developed sternal keel present in *Neuquenornis* (Chiappe & Calvo, 1994). This may suggest ornithuromorphs were not only able to take-off from the ground, but also able to continuously fly for longer distances, which requires continuous power output from a large *M. pectoralis major*. This is consistent with interpretations that Early Cretaceous enantiornithines may be unable to sustain prolonged flight, but prefer to intermittent flight styles such as bounding or flap-gliding flight (Liu et al., 2017; Serrano et al., 2018; Chiappe et al., 2019a; Chiappe et al., 2019b).

The second critical transformation is the formation of the triosseal canal. This is the most complex morphological transformation because of the greater number of elements involved. As the passage of the tendon of the *M. supracoracoideus*, the triosseal canal changes the *M. supracoracoideus* from protractor in early pennaraptorans to elevator of the humerus in ornithothoracines (Novas et al., 2020; Novas et al., 2021b), providing the muscular power for the upstroke during flapping flight. In contrast to extant birds, basal pennaraptorans lack the acrocoracoclavicular joint between the coracoid and furcula and have only two joints between the three pectoral girdle elements at their omal ends (the long synchondrosis coracoscapular joint and the scapuloclavicular joint along the dorsal edge of the acromion of the scapula), which further differ in their morphology compared to extant birds. Hence, the transformation from the pennaraptoran to ornithothoracine condition involved morphological changes in the coracoscapular and the scapuloclavicular joints, as well as the formation of the acrocoracoclavicular joint.

This transformation involved: (1) a reduction in the articular surface between the scapula and coracoid from the long synchondrosis (which may fuse in adults) in oviraptorosaurs (as in *Khaan*) to localized articular surfaces in potential volant dromaeosaurids (as in *Rahonavis*) and avialans (as in *Anchiornis*); (2) cranial migration and enlargement of the acromion process of the scapula, from caudal to the scapula-coracoid articular surface in oviraptorosaurs (*e.g.*, *Khaan* and *Heyuannia*) to elongate and cranially oriented in dromaeosaurids (as in *Bambiraptor*) and avialans crownward of anchiornithids; (3) change in the articulation between the scapula and furcula from contacting along the dorsal edge of the scapular acromion (as in oviraptorosaurs) to the cranial tip of this process (as in paravians) which freed the remainder of the furcular epicleideal process; and (4) the migration of the acrocoracoid process from below the supracoracoid foramen (as in oviraptorosaurs) to above (dorsal) the glenoid fossa (as in ornithothoracines). The last two morphological transformations facilitated the appearance of the acrocoracoclavicular joint between the furcular epicleideal process and the coracoid acrocoracoid process in the Ornithuromorpha. Notably, some of these transformations (*e.g.*, 1, 2) evolved multiple times independently with the repeated evolution of flight in pennaraptorans.

The fully closed, bony triosseal canal was present in at least some early Cretaceous basal ornithuromorphs, such as *Yixianornis,* as indicated by the articular surface for the furcula visible on the acrocoracoid process. Further research on early diverging ornithuromorphs will help clarify the appearance of this feature. However, it is worth noting that a bony, closed triosseal canal may not be necessary to achieve the pulley function of the *M. supracoracoideus* (Novas et al., 2021b; Wang et al., 2022b). Soft tissues like the acrocoraco-acromial ligament may also help to close the supracoracoid canal for the passage of the *M. supracoracoideus* (Ando & Fukata, 2018; Novas et al., 2021b). In this regards, although enantiornithines do not possess a bony closed triosseal canal, the morphology of the pectoral girdle may still permit the *M. supracoracoideus* to function as a pulley as in extant birds (Wang et al., 2022b).

This raises questions as to the function of the additional acrocoracoclavicular joint present in ornithuromorphs, and suggests that the function of the triosseal canal may not be limited to the pulley-like motion of the *M. supracoracoideus*. The acrocoracoclavicular joint in extant birds shows considerable diversity: synovial joint, syndesmosis, and synostosis are all observed (Baumel et al., 1993; Wu et al., 2021b). The significance of this diversity and how it affects avian flight is unclear. During the flight stroke in extant birds, the acrocoracoclavicular joint shows slight displacement together with the movement of the coracoid and the deformation of the furcula (Baier, Gatesy & Dial, 2013), suggesting this joint may play a role in the flight stroke, possibly facilitating the continuous flapping movement in ornithuromorphs. Research focused on the acrocoracoclavicular joint is limited and further analysis of the anatomy, histology and function of this joint will help reveal its role in avialan flight evolution.

In addition to the acrocoracoid process, acromion process and epicleideal process, the procoracoid process is also mentioned as an important component of the triosseal canal (Baumel et al., 1993). *Jeholornis* is the stemward-most appearance of this feature and the only basal bird to possess a procoracoid process (Zheng et al., 2020). Mesozoic ornithuromorphs and the stem-most enantiornithine *Protopteryx* all have procoracoid processes, indicating the procoracoid process evolved at least twice during bird evolution, independently evolving in *Jeholornis* and the common ancestor of ornithothoracines (Zheng et al., 2020). Loss of the procoracoid process in enantiornithines more derived than *Protopteryx* may be secondary, or this feature may have evolved independently in *Protopteryx*. The procoracoid process of certain extant birds is also reduced, such as in the Phasianidae and Passeriformes (Oswald & Steadman, 2015; Wang et al., 2022b). This questions the contribution of the procoracoid process to the triosseal canal and its relation to flight ability. The evolution and secondary loss of the procoracoid process warrants further investigation.

The third major aspect of this transformation is the change in morphology and orientation of the coracoid and the reduction in the angle between the scapula and coracoid. The coracoid elongates from trapezoidal in early pennaraptorans to narrow and strut-like in birds (Turner, Makovicky & Norell, 2012). However, elongation of this element has also evolved independently in other non-avialan volant pennaraptorans and the trapezoidal morphology is retained in the basal birds *Archaeopteryx* and *Sapeornis*. The coracoid body also rotates from craniolaterally oriented in early pennaraptorans to cranially oriented in paravians, with only the lateral margin (caudal margin of more basal pennaraptorans), acrocoracoid process and sternal project visible in lateral view, forming an L-shaped scapulocoracoid (Fig. 1).

During avian flight, the pectoral girdle transmits the lift generated by the wing to the body (Pennycuick, 1967). A strut-like coracoid effectively resists the pressure of the muscles on the thorax when flapping, preventing damage to the thoracic and visceral structures (King & Mclelland, 1984; Gill, 2007), while the coracoid itself experiences great pressure (Samour, 2015). With the same bone mass, narrow strut-like coracoids can have thicker cortical bone compared to the wide and flat axe-like coracoid, permitting this bone to withstand higher pressure without causing damage. This may suggest that elongation of the coracoid indicates flapping, powered flight.

The change in coracoid orientation from lateromedial in early pennaraptorans to dorsoventral in paravians, including non-volant troodontids (Pei et al., 2020), suggests this modification may be related to forelimb and pectoral girdle functions other than powered flight, like sexual display, predation, or nest manipulation. As such, the rotation of the coracoid lay the foundation for the evolution of powered flight in birds and can be considered as an exaptation for flight evolution. The same is true for the cranial migration of the scapuloclavicular joint in paravians (Fig. 1). Alternatively, rotation of the coracoid towards the midline of the body may indicate some form of volant behavior was plesiomorphic to Paraves (or a more inclusive group), as has been previously suggested (Xu et al., 2015; Sullivan, Xu & O’Connor, 2017).

In paravians and oviraptorosaurs with ossified sternal plates, the coracoid articulates in a groove on the cranial margin of the sternum (Burnham, 2004; Zheng et al., 2014; Cau et al., 2021). During powered flight in extant birds, the coracoid moves lateromedially within this groove to assist the flapping movement of the wing (Baier, Gatesy & Dial, 2013; Razmadze, Panyutina & Zelenkov, 2018), whereas in basal pennaraptorans, a groove-like articulation with the sternal plates was likely absent,, such as *Caudipteryx*. This morphological change and rotation of the coracoid coincided with a decrease in the angle formed by the scapula-coracoid from obtuse in early pennaraptorans, to approximately 90° in non-avialan paravians, to an acute angle in basal birds crownward of *Archaeopteryx* (Fig. 1). The acute angle reduces the length of the dorsal elevator muscles(Gill, 2007), therefore decreasing the time required for the muscle to contract, allowing the wing to be uplifted faster. In extant birds, it can be clearly seen that the time required for the upstroke is shorter than the downstroke, which also experiences greater resistance as it pushes against the air (Rayner, 1988; Biewener, 2011). Except for the specialized condition in hummingbirds, the upstroke of extant birds does not generate lift (Biewener, 2011; Chin & Lentink, 2019), thus reduction of the time required to make the upstroke is beneficial to reduce altitude loss during powered flight.

It is proposed that flight capacity originated multiple times in pennaraptors (Wang et al., 2019; Pei et al., 2020), especially in paravians, accompanied by occurrence of flight adaptive features in different clades. Several avialian-like features are found in species of Unenlagiinae, Microraptorinae and the taxon *Bambiraptor*, which is considered to belong to the Saurornitholestinae, such as developed acromion process of scapula, reduced articular surface of coracoscapular joint and lateral facing glenoid fossa. The occurrence of these features in several dromaeosaurid clades suggests that these features may represent the plesiomorphic dromaeosaurid condition, which evolved independently from those similar features in avialans.

The existence of these avialan-like plesiomorphic dromaeosaurid conditions is consistent with the evolution of powered flight or nearly powered flight capacity in several dromaeosaurid clades. This may support the alternative hypothesis that flight was plesiomorphic to Paraves and secondarily lost, rather than evolved repeatedly or explain the repeated evolution of flight in dromaeosaurids, because their plesiomorphic morphology provided the framework for potential flight. Additionally, in microraptorines and *Bambiraptor*, the narrow neck of the coracoid differs from the axe-like coracoid of early pennaraptorans, but represents an intermediate morphology with the strut-like avian-like coracoid.

However, these volant dromaeosaurids still lack some important flight adaptions of the pectoral girdle that are found in ornithothoracines, such as the strut-like coracoid, dorsolaterally oriented glenoid fossa, and well-developed acrocoracoid process dorsal than the glenoid fossa, suggesting that if they were volant, they had limited powered flight capacity compared to ornithothoracine birds. Their wings may have functioned to parachute from heights and glide short distances, but ground takeoff and long distance continuous flight would have been challenging to these possibly volant dromaeosaurids, such as the microraptorines.

In avialans, the morphology of the pectoral girdle diversifies (*e.g.*, the different coracoid morphologies) as a product of the parallel refinement of the flight apparatus following the rapid diversification of avialan lineages in the latest Jurassic. A modern-like pressure transition system (strut-like coracoid) and glenoid fossa orientation characterizes the Ornithothoraces. The acrocoracoclavicular joint evolved in the Ornithuromorpha and indicates the earliest appearance of a fully closed bony triosseal canal. These transformations suggest that, although flight may have evolved several times in theropods, flight capabilities comparable to living birds were restricted to the Ornithuromorpha. The loss of some features (*e.g.*, the acute angle between scapula and coracoid, well-developed acromion of the scapula and acrocoracoid process of the coracoid, as well as the triosseal canal) in secondarily flightless birds, both Mesozoic and extant, further support inferences that these features are intrinsically linked to the evolution and refinement of pennaraptoran flight.

## Conclusions

Through the comparative analysis of pennaraptoran shoulder girdle characteristics, this study summarizes the major transformations of the pectoral girdle that appeared during the evolution of pennaraptoran flight. These include changes in the orientation and position of the glenoid fossa, in the orientation and shape of the coracoid and the angle between the scapula and coracoid, the relative position of the acrocoracoid process of the coracoid, the articulation between the acromion of the scapula and the epicleideal process of the furcula, and the formation of the triosseal canal in birds. The three joints that link these pectoral girdle elements all are modified, including the morphology and position of the scapula-furcula joint, the morphology and type of the scapula-coracoid joint, and the formation of a new joint between the furcula and the coracoid.

Some character changes precede the rise of birds, but appear to be linked to the appearance of flight potential and the possible independent evolution of volant behavior in some non-avialan pennaraptorans. The morphology of the pectoral girdle elements becomes further diversified in avialans, indicating different parallel attempts to refine flight performance in early birds from a flight apparatus tentatively limited to gliding or weak flapping flight, as in *Archaeopteryx*, to powered flight in ornithothoracines and possibly also *Jeholornis*. The glenoid fossa of the ornithothoracines could support similar forelimb flapping movement as observed in extant birds, and marks the appearance of a new joint between the coracoid and furcula, suggesting this clade had obtained fully equipped powered flight by the Early Cretaceous, only 20 million years after the appearance of the oldest known probable bird *Archaeopteryx*. The function of these morphological changes have been studied for decades as discussed here, yet some characteristics remain poorly understood. It is still unclear what is the functional difference between a fused and separate scapula-coracoid joint, or between the fully formed triosseal canal of ornithuromorphs and that open canal present in enantiornithines. Additional fossil material and greater availability of 3D CT data will hopefully lend answers to these questions in the future.

## Supplemental Information

10.7717/peerj.16960/supp-1 Supplemental Information 1 Non-nestling specimens of non-avialian pennaraptorans and anchiornithids that preserved pectoral girdle elements

## Additional Information and Declarations

### Data Availability

The following information was supplied regarding data availability: This is a literature review.
