# Supplementary material for: Transformation of the pectoral girdle in pennaraptorans: critical steps in the formation of the modern avian shoulder joint
Source: PeerJ. 2024 Feb 29;12:e16960. doi: 10.7717/peerj.16960 (PMC10909347; doi:10.7717/peerj.16960)
Supplement: Supplemental Information 1 [file peerj-12-16960-s001.pdf]

SUPPLYMENTRAY INFORMATION

Transformation of the pectoral girdle in pennaraptorans: critical steps in the formation of the modern avian shoulder joint

Wu et al.

This PDF file includes
Supplementary Table 1
Supplementary Figures 1 to 6
Supplementary References

Table S1. Non-nestling specimens of non-avialian pennaraptorans and anchiornithids that preserved pectoral girdle elements.

#	*species*	collection number	ontogeny stage[1]	pectoral girdle preservation	Scapula-coracoid joint[2]	glenoid orintation	scapula-coracoid angle	reference
1	*Oviraptor philoceratops*	IGM 100/42; 100/36	not mention	scapula, coracoid, furcula	synchondrosis	caudoventrally	?	(Barsbold, 1983)
2	*Caudipteryx zoui*	NGMC 97-4	not mention	scapula, coracoid, furcula	separated	?	?	(Ji et al., 1998)
		IVPP V12430	Adult	scapula, coracoid	separated	?	> 90°	(Zhou and Wang, 2000)
		BPM 0001	Adult	scapula, coracoid, ?furcula	fused	?	90°	(Zhou et al., 2000)
3	*Avimimus portentosus*	PIN 3907-1	Adult	scapula, coracoid,	fused	caudoventrally	160°	(Kurzanov, 1981)
4	*Avimimus nemegtensis*	MPC-NEE.2016-257	Adult	scapula, coracoid,	fused	caudoventrally	?	(Funston et al., 2018)
5	*Tongtianlong limosus*	DYM-2013-8;	not mention	scapula, coracoid, furcula	separated	?	130°	(Lü et al., 2016)
6	*Microvenator celer*	AMNH 3041; YPM 5366	juvenile	coracoid	synchondrosis	caudoventrally	?	(Makovicky and Sues, 1998)
7	*Chirostenotes pergracilis*	TMP 1979.020.0001	juvenile or subadult	coracoid	separated	caudoventrally	?	(Funston, 2020)
8	*Rinchenia mongoliensis*	MPC-D 100/32A	juvenile or subadult	scapula, coracoid, furcula	synchondrosis	caudoventrally	?	(Funston et al., 2018)
9	*Elmisaurus rarus*	MPC-D 102/113	not mention	scapula, coracoid,	separated	more caudally oriented	?	(Funston et al., 2021)
10	*Ningyuansaurus wangi*	no specimen number mentioned	not mention	scapula, coracoid	separated	?	?	(Ji et al., 2012)
11	*Heyuannia yanshini*	MPC-D 100/30, 100/33	not mention	scapula, coracoid, furucla	synchondrosis	caudoventrally	> 90°	(Barsbold, 1981, 1983; Osmólska et al., 2007; Funston et al., 2018)
12	*Heyuannia huangi*	HYMV1-2, 1-5	not mention	scapula, incomplete coracoid, furcula	fused	laterally	145°	(Lü, 2003; Lü et al., 2005b)

13	*Nemegtomaia barsboldi*	GIN 100/2112; MPC-D 107/15	not mention	partly scapula	?	?	?	(Lü et al., 2004, 2005a; Fanti et al., 2012)
14	*Citipati osmolskae*	IGM 100/978; IGM 100/979	adult	scapula, coracoid, furcula	fused	?	?	(Clark et al., 1999, 2001)
15	*Similicaudipteryx yixianensis*	IVPP V12556	not mention	part scapula, coracoid	synchondrosis	?	?	(He et al., 2008)
16	*Xingtianosaurus ganqi*	IVPP V13390	not mention	scapula	?	?	?	(Qiu et al., 2019)
17	*Gigantorapter erlianensis*	LH V0011	young adult	scapula	?	?	?	(Xu et al., 2007)
18	*Anzu wyliei*	CM 78000; 78001; MRF 319	not mention	scapula, coracoid	fused	caudoventrally	?	(Lamanna et al., 2014)
19	*Apatoraptor pennatus*	TMP 1993.051.0001	relatively mature individuals	scapula, coracoid	synchondrosis	laterally	?	(Funston and Currie, 2016)
20	*Nankangia jiangxiensis*	GMNH F10003	not mention	scapula,coracoid, part furcula	fused	caudoventrally	?	(Lü et al., 2013b)
21	*Gobiraptor minutus*	MPC-D 102/111	very early ontogeny stage	scapula	separated	ventrally	?	(Lee et al., 2019)
22	*Jiangxisaurus ganzhouensis*	HGM41-HIII0421	juvenile or subadult	scapula, coracoid, furcula	separated	?	?	(Wei et al., 2013)
23	*Khaan mckennai*	IGM 100/1127; IGM 100/1002	not mention	scapula, coracoid, furcula	fused	laterally	150°-160°	(Clark et al., 2001; Balanoff and Norell, 2012)
24	*Oksoko avarsan*	MPC-D 102/110	juvenile	scapula, coracoid, furcula	synchondrosis	caudoventrally	?	(Funston et al., 2020)f
25	*Wulatelong gobiensis*	IVPP V 18409	adult	scapula, coracoid,	fused	not preserve	>90°	(Xu et al., 2013b)
26	*Yulong mini*	HGM 41HIII-0107	juvenile	scapula, coracoid, furcula	synchondrosis	caudoventrally	?	(Lü et al., 2013a)
27	*Conchoraptor gracilis*	IGM 100/3006	not mention	not mention	unfused	?	?	(Balanoff and Norell, 2012)
28	*Epidexipteryx hui*	IVPP V15471	subadult	scapula, coracoid	separated	?	?	(Zhang et al., 2008)
29	*Ambopteryx longibrachium*	IVPP V24192	subadult or adult	scapula, coracoid	separated	?	?	(Wang et al., 2019)

30	*Scansoriopteryx heilmanni*	CAGS02-IG-gausa-1/DM 607.	nestling 2 to 3 weeks	part scapula, coracoid and clavicle	separated	?	?	(Czerkas and Yuan, 2002)
31	*Yi qi*	STM 31-2	Adult	scapula	?	?	?	(Xu et al., 2015)
32	*Epiderdrosaurus ningchengensis*	IVPP V12653	juvenile	scapula, coracoid	separated	?	?	(Zhang et al., 2002)
33	*Zhongornis haoae*	DNHM D2456;DNHM D2455	juvenile	scapula, coracoid, furcula	separated	?	?	(Gao et al., 2008)
34	*Overoraptor chimentoi*	MPCA-Pv 805	Not metion	only part scapula preserve	?	?	?	(Motta et al., 2020)
35	*Pneumatoraptor fodori*	V.2008.38.1.	adult	scapula, coracoid	fused	?	?	(Ősi et al., 2010)
36	*Hesperornithoides miessleri*	WYDICE-DML-001	Adult or subadult	part scapula, coracoid	?	?		(Hartman et al., 2019)
37	*Jianianhualong tengi*	DLXH 1218	Adult	scapula, coracoid	separate	?	?	(Xu et al., 2017a)
38	*Mei long*	IVPP V12733	juvenile	scapula, coracoid, furcula	separated	?	?	(Xu et al., 2004)
		DNHM D2154	not mention	scapula, coracoid, ?furcula	maybe fused	ventrally	90°	(Gao et al., 2012)
39	*Gobivenator mongoliensis*	MPC-D 100/86	subadult	scapula, coracoid	separated	caudoventrally	?	(Tsuihiji et al., 2014)
40	*Jinfengopteryx elegans*	CAGS-IG-04-0801	not mention	scapula, coracoid	fused	?	90°	(Ji et al., 2005)
41	*Linhevenator tani*	LHV0021	adult	scapula	?	ventrally	?	(Xu et al., 2011a)
42	*Sinovenator changii*	IVPP V12615, 12583	not mention	scapula, coracoid	synchondrosis	laterraly	?	(Zhang et al., 2002) wang et al., 2022 under review
43	*Liaoningvenator curriei*	DNHM D3012	subadult	scapula, coracoid	synchondrosis	ventrally	< 90°	(Shen et al., 2017)
44	*Sinornithoides youngi*	IVPP V 9612	immutre	scapula, coracoid, furcula	synchondrosis	?	?	(Russell and Dong, 1993; Currie and Dong, 2001)
45	*Troodon formosus*	University Alberta Collections	not mention	scapula, part coracoid	synchondrosis	?	?	(Gilmore, 1924)
46	*Yixianosaurus longimanus*	IVPP V12638	subadult	scapula, coracoid	synchondrosis	?	?	(Xu and Wang, 2003; Xu et al., 2013a)
47	*Rahonavis ostromi*	UA 8656	mature adult	scapula	separate	laterally	?	(Forster et al., 1998)

48	*Buitreraptor gonzalezorum*	MPCN-PV-598	subadult	scapula, coracoid, furcula	synchondrosis	laterally	?	(Novas et al., 2018)
		MPCA 245	adult	scapula, coracoid, furcula	synchondrosis	?	?	(Gianechini et al., 2018)
49	*Unenlagia comahuensis*	MCF PVPH 78	not mention	scapula	separate	laterally	?	(Novas and Puerta, 1997)
50	*Velociraptor mongoliensis*	IGM 100/976, 982, 985, 986	not mention	scapula, coracoid, furcula	fused	laterally	?	(Norell et al., 1997; Parsons and Parsons, 2009)
51	*Bambiraptor feinbergi*	AMNH FR 30554	juvenile	scapula, coracoid, furcula	synchondrosis	laterally	?	(Burnham et al., 2000; Burnham, 2004)
52	*Sinornithosaurus millenii*	IVPP V12811	Adult	scapula, coracoid, furcula	synchondrosis	laterally	< 90°	(Xu et al., 1999)
53	*Microraptor zhaoianus*	CAGS 20-7-004 and 20-8-001	adult	scapula, coracoid	fused	?	105°	(Hwang et al., 2002)
54	*Microraptor hangqingi*	LVH 0026	not mention	scapula, coracoid, furcula	fused	laterally	90°	(Gong et al., 2012)
55	*Microraptor gui*	IVPP V13352(holotype), 13320, 13351, TNP00996	not mention	scapula, coracoid, furcula	fused	laterally	< 90°	(Xu et al., 2003)
56	*Wulong bohaiensis*	D2933	juvenile	scapula, coracoid, furcula	synchondrosis	?	?	(Poust, 2014; Poust et al., 2020)
57	*Tianyuraptor ostromi*	STM 1-3	subadult	scapula, coracoid, furcula	separeted	laterally	?	(Zheng et al., 2010)
58	*Changyuraptor yangi*	HG B016	not mention	scapula, furcula	?	?	?	(Han et al., 2014)
59	*Zhenyuanlong suni*	JPM-0008	not mention	scapula, coracoid	?	?	?	(Lü and Brusatte, 2015)
60	*Zhongjianosaurus yangi*	IVPP V22775	not mention	scapula, coracoid, furcula	fused	laterally	~90°	(Xu et al., 2017b)
61	*Luanchuanraptor henanensis*	41HIII-0100	not mention	scapula, coracoid	fused	caudoventrally	?	(Lü et al., 2007)
62	*Balaur bondoc*	EME PV.313	Adult	scapula, coracoid	fused	ventrally lateral	?	(Csiki et al., 2010; Brusatte et al., 2013)
63	*Deinonychus antirrhopus*	MCZ 8791	juvenile	scapula, coracoid	synchondrosis	?	?	(Parsons and Parsons, 2009)
		OMNH 50268	subadult	scapula, coracoid	synchondrosis	?	?	(Brinkman et al., 1998)

		AMNH 3015, YPM 5236	Adult	scapula, coracoid	fused	anteroventrally with lateral position	~90°	(Ostrom, 1969, 1974; Gishlick, 2001; Parsons and Parsons, 2009)
64	*Achillobator giganticus*	FR. MNUFR-15	not mention	scapula	synchondrosis	ventrolaterally	?	(Perle et al., 1999)
65	*Linheraptor exquisitus*	IVPP V 16923	Adult	only scapula preserve	?	?	?	(Xu et al., 2010)
66	*Anchiornis huxleyi*	IVPP V14378	subadult or young adult	scapula, coracoid, furcula	?	?	?	(Xu et al., 2009)
		BMNHC PH804, BMNHC PH822	not mention	scapula, coracoid, furcula	synchondrosis	?	?	(Pei et al., 2017)
		41 HIII 0404, 0415	probably Adult	scapula, coracoid, furcula	synchondrosis	laterally	?	(Guo et al., 2018)
		LPM-B00169	not mention	scapula, coracoid, furcula	fused	?	?	(Hu et al., 2009)
67	*Aurornis xui*	YFGP-T5198	probably Adult	scapula, coracoid, furcula	separate	?	?	(Godefroit et al., 2013a)
68	*Eosinopteryx brevipenna*	YFGP-T5197	subadult or adult	scapula, coracoid, part furcula	synchondrosis	?	?	(Godefroit et al., 2013b)
69	*Xiaotingia zhengi*	STM 27-2	probably Adult	scapula, coracoid, furcula	separate	?	?	(Xu et al., 2011b)
70	*Serikornis sungei*	PMOL-AB00200	subadult	scapula, coracoid, furcula	synchondrosis	?	?	(Lefèvre et al., 2017)
71	*Caihong juji*	PMoL-B00175	probably Adult	scapula, coracoid, furcula	?	?	?	(Hu et al., 2018)

1. Based on the original evaluation in the referenced text.
2. The joint between scapula and coracoid are re-evaluated based on the description and figures in the original text. The original descriptions include suture, tightly articulated, separated, unfused. Sutured and tightly articulated are replaced by synchondrosis here (see explanation in the text). Separate here means the two elements are separately preserved with clear gap, or smooth articular surface on each side. It is possible that there are soft tissues connecting these two elements when the animal was alive, but without further evidence to support the present of certain soft tissue we prefer to be conservative and keep the original description. "Unfused" could either be "synchondrosis" or "separate", so we further classified those description according to the figures or other available information.

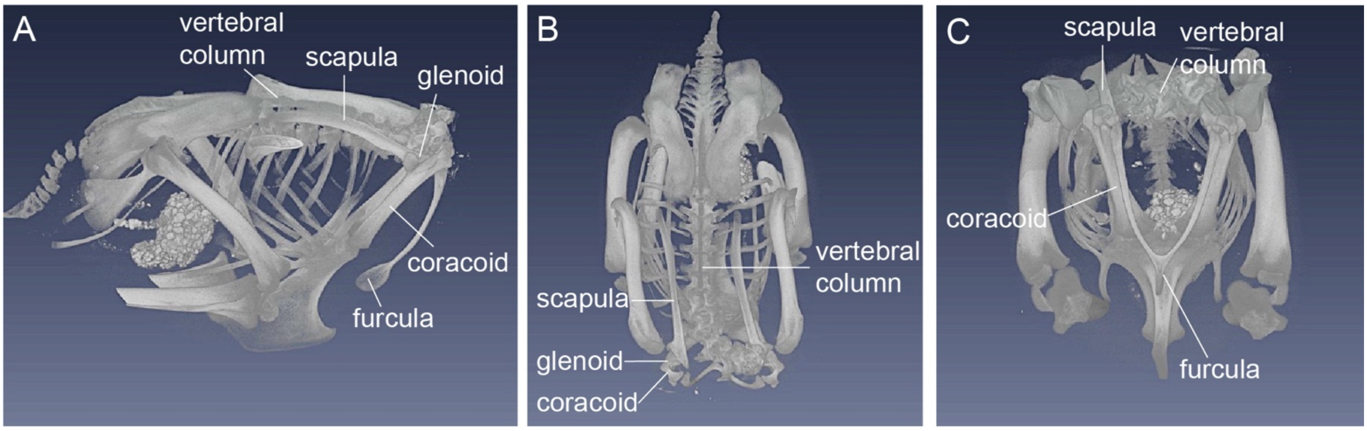

Supplementary Figure 1. 3D reconstruction of domestic chicken skeleton in lateral (A), dorsal(B) and cranial (C) view, showing the scapula located at the dorsal side of the thoracic cage, the external surface of the coracoid ventrally oriented, and the glenoid dorsolaterally oriented. The specimens was bought from the market and CT scaned in IVPP.

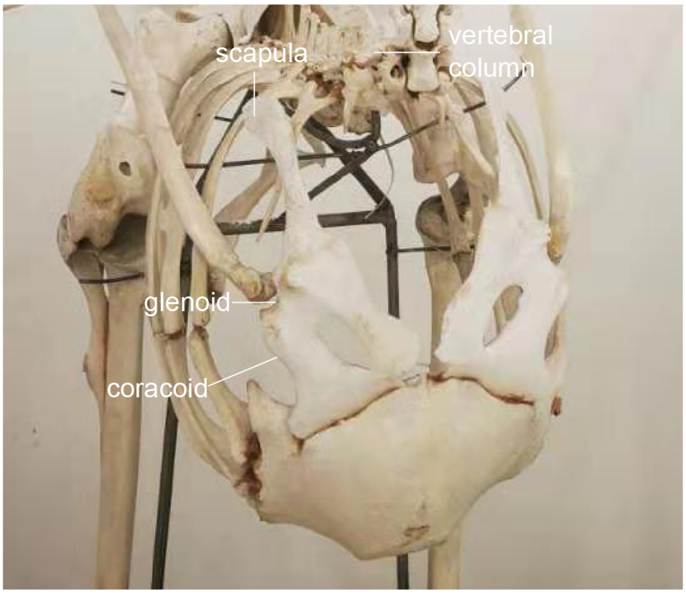

Supplementary Figure 2. Common ostrich skeleton (IVPP OV586) in craniolateral view, showing the scapula located at the lateral side of the thoracic cage, external surface of the coracoid cranially oriented and the lateral surface facing laterally. Photo taken by the author.

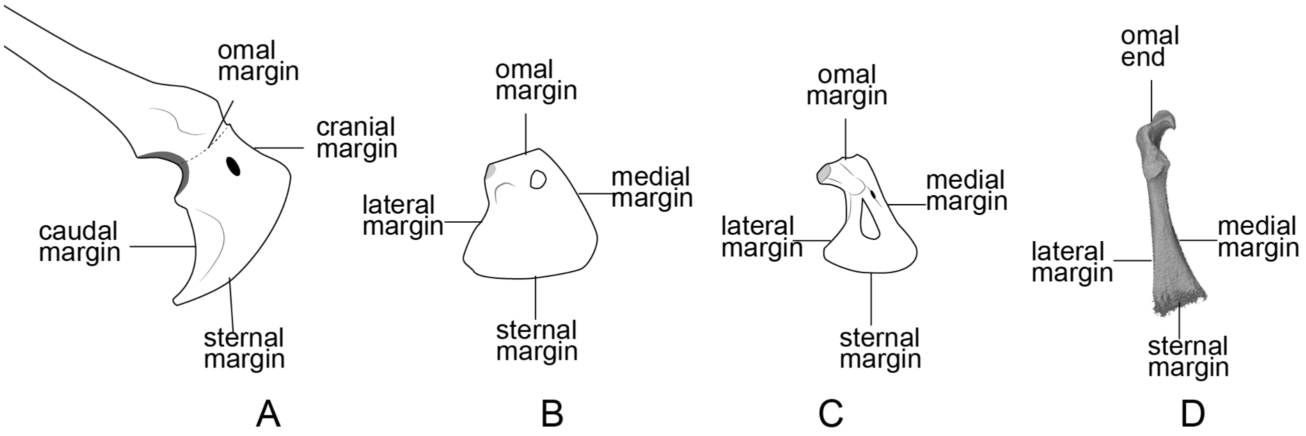

Supplementary Figure 3. Comparison of the Coracoid of selected pennaraptorans. A, *Heyuannia* (oviraptorosaur) in lateral view based on MPC-D 100/30 modified after Osmólska et al., 2007; B, *Sinovenator* (troodontids) in cranial view based on IVPP V12615 after Xu et al., 2002; C, *Sinornithosaurus* (microraptorine) in cranial view based on IVPP V12811 modified from Xu et al., 1999, D, 3D reconstruction from CT scan of domestic chick in dorsal view.

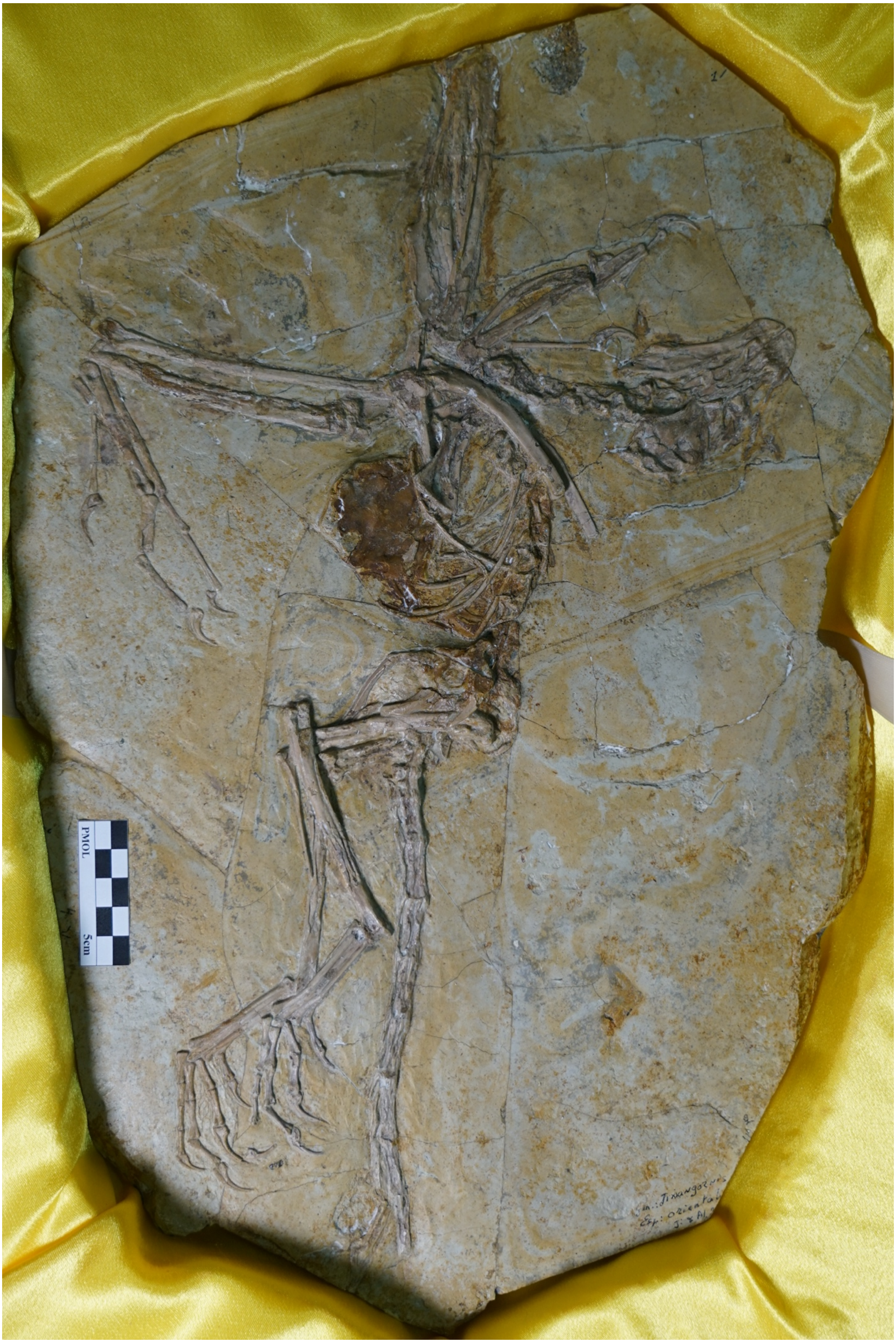

Supplementary Figure 4. Photograph of *Jeholornis* YFGP-yb2.

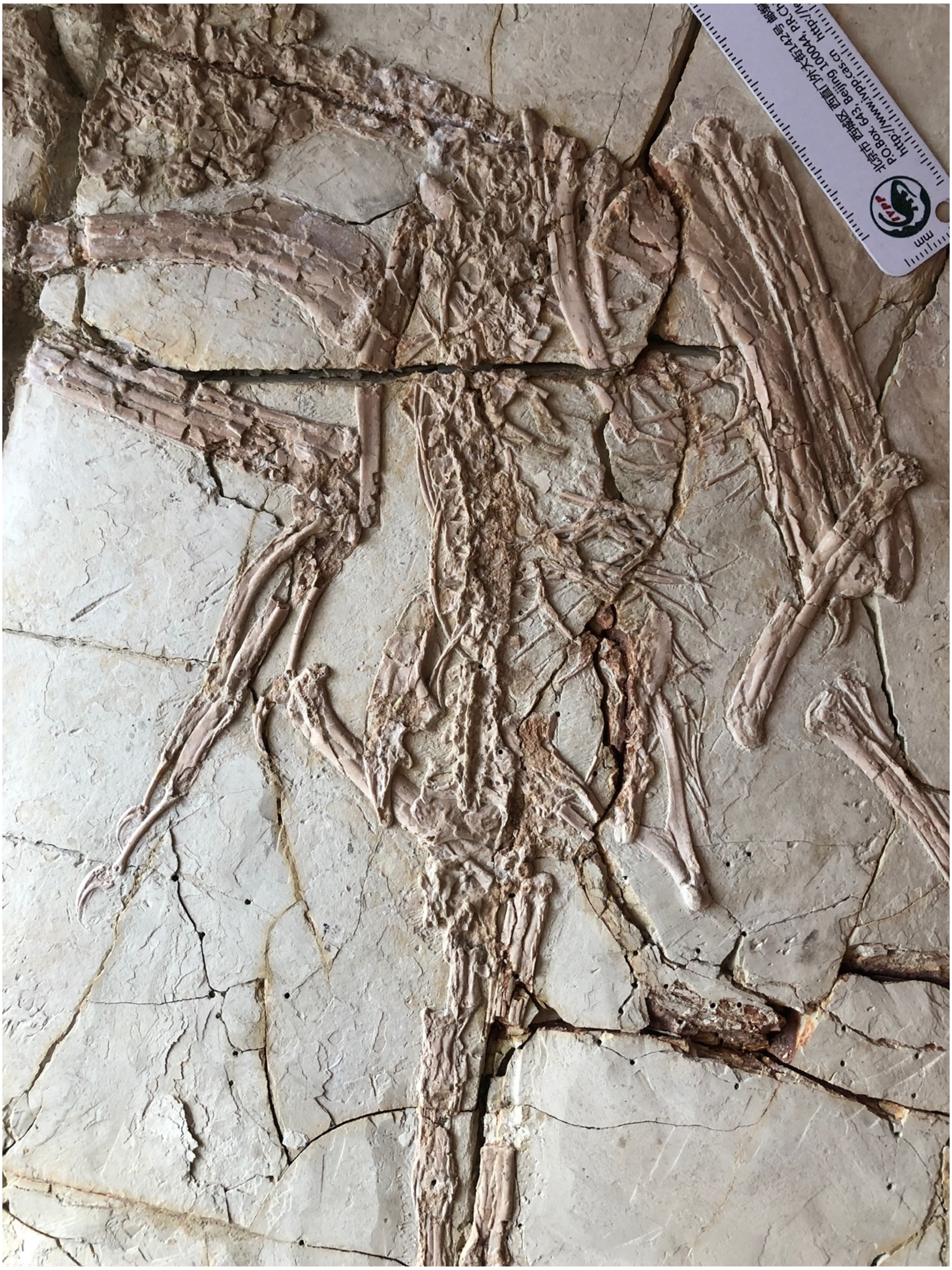

Supplementary Figure 5. Photograph of *Jeholornis* IVPP V 13353

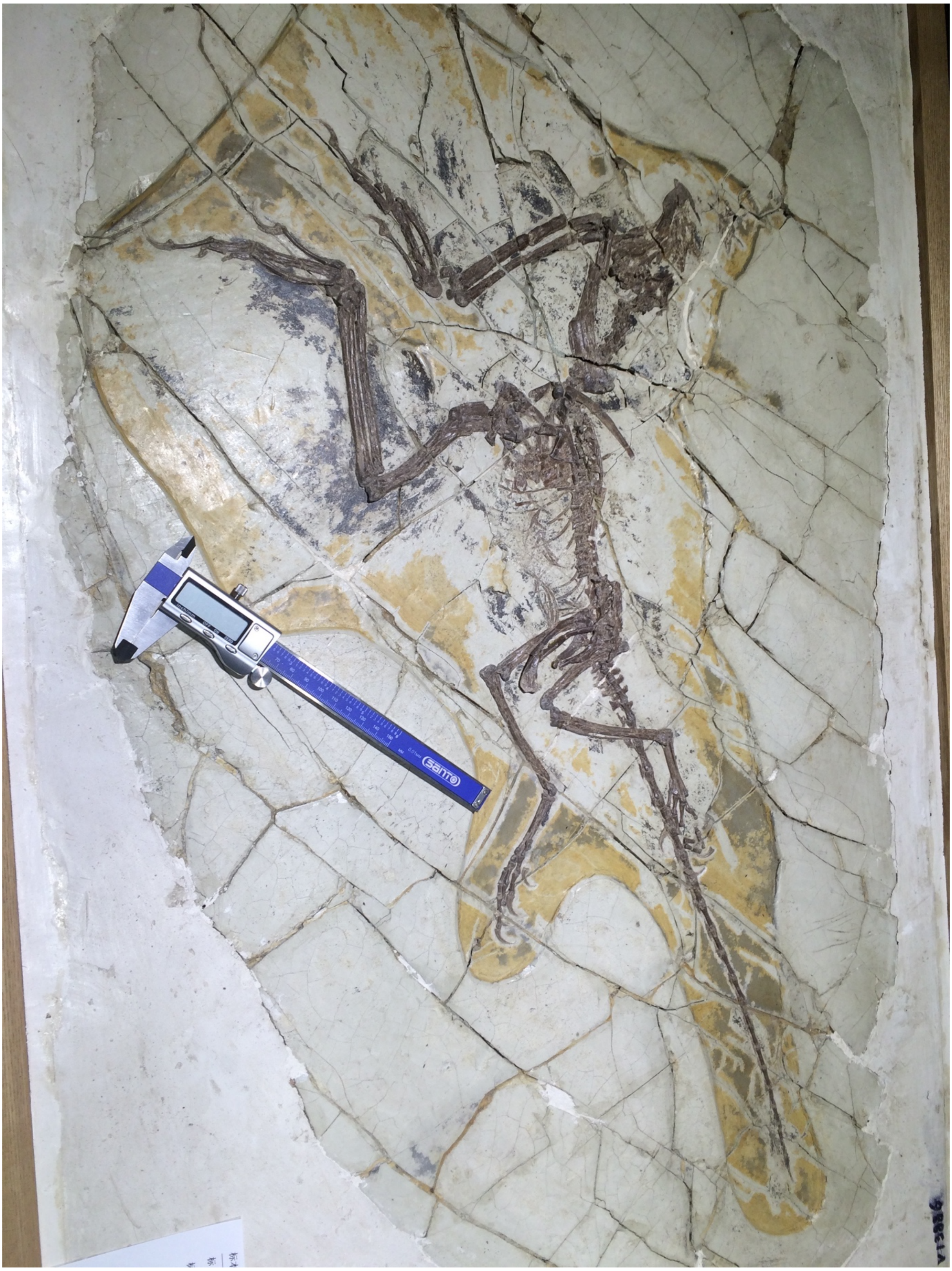

Supplementary Figure 6. Photograph of *Jeholornis* IVPP V 13886

Reference of Table S1 :

Balanoff, A. M., and Norell, M. A. (2012). Osteology of *Khaan mckennai* (Oviraptorosauria: Theropoda). *Bull. Am. Mus. Nat. Hist.* 2012, 1–77. doi:10.1206/803.1.

Barsbold, R. (1981). Toothless carnivorous dinosaurs of Mongolia. *Tr. Sovmest. Sov.-Mongol'skoi Paleontol. Ekspeditsii* 15, 28–39.

Barsbold, R. (1983). "Avian" features in the morphology of predatory dinosaurs. *Trans. Jt. Sov.-Mong. Paleontol. Exped.* 24, 96–103.

Brinkman, D. L., Cifelli, R. L., and Czaplewski, N. J. (1998). *First Occurrence of Deinonychus Antirrhopus (Dinosauria: Theropoda) from the Antlers Formation (Lower Cretaceous, Aptian-Albian) of Oklahoma*. University of Oklahoma.

Brusatte, S. L., Vremir, M., Csiki-Sava, Z., Turner, A. H., Watanabe, A., Erickson, G. M., et al. (2013). The osteology of *Balaur bondoc*, an island-dwelling dromaeosaurid (Dinosauria: Theropoda) from the Late Cretaceous of Romania. *Bull. Am. Mus. Nat. Hist.* 2013, 1–100. doi:10.1206/798.1.

Burnham, D. A. (2004). New Information on *Bambiraptor feinbergi* (Theropoda: Dromaeosauridae) from the Late Cretaceous of. *Feather. Dragons Stud. Transit. Dinosaurs Birds*, 67.

Burnham, D. A., Derstler, K. L., Currie, P. J., Bakker, R. T., Zhou, Z., and Ostrom, J. H. (2000). Remarkable new birdlike dinosaur (Theropoda: Maniraptora) from the Upper Cretaceous of Montana. *Paleontol. Contrib. New Ser. 1992-2009*. doi:10.17161/PCNS.1808.3761.

Clark, J. M., Norell, M. A., and Barsbold, R. (2001). Two new oviraptorids (Theropoda: Oviraptorosauria), Upper Cretaceous Djadokhta Formation, Ukhaa Tolgod, Mongolia. *J. Vertebr. Paleontol.* 21, 209–213. doi:10.1671/0272-4634(2001)021[0209:TNOTOU]2.0.CO;2.

Clark, J. M., Norell, M. A., and Chiappe, L. M. (1999). An oviraptorid skeleton from the late Cretaceous of Ukhaa Tolgod, Mongolia, preserved in an avianlike brooding position over an oviraptorid nest. *Am. Mus. Novit.*, 1–36.

Csiki, Z., Vremir, M., Brusatte, S. L., and Norell, M. A. (2010). An aberrant island-dwelling theropod dinosaur from the Late Cretaceous of Romania. *Proc. Natl. Acad. Sci.* 107, 15357–15361. doi:10.1073/pnas.1006970107.

Currie, P. J., and Dong, Z. (2001). New information on Cretaceous troodontids (Dinosauria, Theropoda) from the People's Republic of China. *Can. J. Earth Sci.* 38, 1753–1766. doi:10.1139/e01-065.

Czerkas, S. A., and Yuan, C. (2002). An arboreal maniraptoran from northeast China. *Dinosaur Mus. J.* 1, 63–95.

Fanti, F., Currie, P. J., and Badamgarav, D. (2012). New specimens of Nemegtomaia from the Baruungoyot and Nemegt formations (Late Cretaceous) of Mongolia. *PLoS One* 7, e31330. doi:10.1371/journal.pone.0031330.

Forster, C. A., Sampson, S. D., Chiappe, L. M., and Krause, D. W. (1998). The Theropod Ancestry of Birds: New Evidence from the Late Cretaceous of Madagascar. *Science* 279, 1915–1919. doi:10.1126/science.279.5358.1915.

Funston, G. (2020). Caenagnathids of the Dinosaur Park Formation (Campanian) of Alberta, Canada: anatomy, osteohistology, taxonomy, and evolution. *Vertebr. Anat. Morphol. Palaeontol.* 8, 105–153. doi:10.18435/vamp29362.

Funston, G. F., Chinzorig, T., Tsogtbaatar, K., Kobayashi, Y., Sullivan, C., and Currie, P. J. (2020). A new two-fingered dinosaur sheds light on the radiation of Oviraptorosauria. *R. Soc. Open Sci.* 7, 201184. doi:10.1098/rsos.201184.

Funston, G. F., and Currie, P. J. (2016). A new caenagnathid (Dinosauria: Oviraptorosauria) from the Horseshoe Canyon Formation of Alberta, Canada, and a reevaluation of the relationships of Caenagnathidae. *J. Vertebr. Paleontol.* 36, e1160910. doi:10.1080/02724634.2016.1160910.

Funston, G. F., Currie, P. J., Tsogtbaatar, C., and Khishigjav, T. (2021). A partial oviraptorosaur skeleton suggests low caenagnathid diversity in the Late Cretaceous Nemegt Formation of Mongolia. *PLOS ONE* 16, e0254564. doi:10.1371/journal.pone.0254564.

Funston, G. F., Mendonca, S. E., Currie, P. J., and Barsbold, R. (2018). Oviraptorosaur anatomy, diversity and ecology in the Nemegt Basin. *Palaeogeogr. Palaeoclimatol. Palaeoecol.* 494, 101–120. doi:10.1016/j.palaeo.2017.10.023.

Gao, C., Chiappe, L. M., Meng, Q., O'connor, J. K., Wang, X., Cheng, X., et al. (2008). A New Basal Lineage of Early Cretaceous Birds from China and Its Implications on the Evolution of the Avian Tail. *Palaeontology* 51, 775–791. doi:10.1111/j.1475-4983.2008.00793.x.

Gao, C., Morschhauser, E. M., Varricchio, D. J., Liu, J., and Zhao, B. (2012). A Second Soundly Sleeping Dragon: New Anatomical Details of the Chinese Troodontid Mei long with Implications for Phylogeny and Taphonomy. *PLoS ONE* 7. doi:10.1371/journal.pone.0045203.

Gianechini, F. A., Makovicky, P. J., Apesteguía, S., and Cerda, I. (2018). Postcranial skeletal anatomy of the holotype and referred specimens of Buitreraptor gonzalezorum Makovicky, Apesteguía and Agnolín 2005 (Theropoda, Dromaeosauridae), from the Late Cretaceous of Patagonia. *PeerJ* 6, e4558. doi:10.7717/peerj.4558.

Gilmore, C. W. (1924). *On* Troodon *validus: An Orthopodous Dinosaur from the Belly River Cretaceous of Alberta, Canada*. University of Alberta Press.

Gishlick, A. D. (2001). "The function of the manus and forelimb of Deinonychus antirrhopus and its importance for the origin of avian flight," in *New Perspectives on the Origin and Early Evolution of Birds*, eds. J. Gauthier and L. F. Gall (New Haven: Peabody Museum of Natural History), 301–318.

Godefroit, P., Cau, A., Dong-Yu, H., Escuillié, F., Wenhao, W., and Dyke, G. (2013a). A Jurassic avialan dinosaur from China resolves the early phylogenetic history of birds. *Nature* 498, 359–362. doi:10.1038/nature12168.

Godefroit, P., Demuynck, H., Dyke, G., Hu, D., Escuillié, F., and Claeys, P. (2013b). Reduced plumage and flight ability of a new Jurassic paravian theropod from China. *Nat. Commun.* 4, 1394. doi:10.1038/ncomms2389.

Gong, E.-P., Martin, L. D., Burnham, D. A., Falk, A. R., and Hou, L.-H. (2012). A new species of Microraptor from the Jehol Biota of northeastern China. *Palaeoworld* 21, 81–91. doi:10.1016/j.palwor.2012.05.003.

Guo, X., Xu, L., and Jia, S. (2018). Morphological and Phylogenetic Study Based on New Materials of *Anchiornis huxleyi* (Dinosauria, Theropoda) from Jianchang, Western Liaoning, China. *Acta Geol. Sin. - Engl. Ed.* 92, 1–15. doi:10.1111/1755-6724.13491.

Han, G., Chiappe, L. M., Ji, S.-A., Habib, M., Turner, A. H., Chinsamy, A., et al. (2014). A new raptorial dinosaur with exceptionally long feathering provides insights into dromaeosaurid flight performance. *Nat. Commun.* 5, 4382. doi:10.1038/ncomms5382.

Hartman, S., Mortimer, M., Wahl, W. R., Lomax, D. R., Lippincott, J., and Lovelace, D. M. (2019). A new paravian dinosaur from the Late Jurassic of North America supports a late acquisition of avian flight. *PeerJ* 7, e7247. doi:10.7717/peerj.7247.

He, T., Wang, X., and Zhou, Z. (2008). A new genus and species of caudipterid dinosaur from the Lower Cretaceous Jiufotang

Formation of western Liaoning, China. *Vertebr. Palasiat.* 46, 178.

Hu, D., Clarke, J. A., Eliason, C. M., Qiu, R., Li, Q., Shawkey, M. D., et al. (2018). A bony-crested Jurassic dinosaur with evidence of iridescent plumage highlights complexity in early paravian evolution. *Nat. Commun.* 9. doi:10.1038/s41467-017-02515-y.

Hu, D., Hou, L., Zhang, L., and Xu, X. (2009). A pre-*Archaeopteryx* troodontid theropod from China with long feathers on the metatarsus. *Nature* 461, 640–643. doi:10.1038/nature08322.

Hwang, S. H., Norell, M. A., Ji, Q., and Gao, Keqin. (2002). New Specimens of *Microraptor zhaoianus* (Theropoda: Dromaeosauridae) from Northeastern China. *Am. Mus. Novit.* 3381, 1–44. doi:10.1206/0003-0082(2002)381<0001:NSOMZT>2.0.CO;2.

Ji, Q., Currie, P. J., Norell, M. A., and Ji, S.-A. (1998). Two feathered dinosaurs from northeastern China. *Nature* 393, 753–761.

Ji, Q., Ji, S.-A., Lü, J.-C., You, H.-L., Chen, W., Liu, Y.-Q., et al. (2005). First avialian bird from China. *Geol. Bull. China* 24, 197–210.

Ji, Q., Lü, J. C., Wei, X. F., and Wang, X. R. (2012). A new oviraptorosaur from the Yixian Formation of Jianchang, western Liaoning Province, China. *Geol. Bull. China* 31, 2102–2107.

Kurzanov, S. M. (1981). An unusual theropod from the Upper Cretaceous of Mongolia. *Jt. Sov.-Mong. Paleontol. Exped.* 15, 39–49.

Lamanna, M. C., Sues, H.-D., Schachner, E. R., and Lyson, T. R. (2014). A new large-bodied oviraptorosaurian theropod dinosaur from the latest Cretaceous of western North America. *PloS One* 9, e92022. doi:10.1371/journal.pone.0092022.

Lee, S., Lee, Y.-N., Chinsamy, A., Lü, J., Barsbold, R., and Tsogtbaatar, K. (2019). A new baby oviraptorid dinosaur (Dinosauria: Theropoda) from the Upper Cretaceous Nemegt Formation of Mongolia. *PLoS ONE* 14, e0210867. doi:10.1371/journal.pone.0210867.

Lefèvre, U., Cau, A., Cincotta, A., Hu, D., Chinsamy, A., Escuillié, F., et al. (2017). A new Jurassic theropod from China documents a transitional step in the macrostructure of feathers. *Sci. Nat.* 104, 74. doi:10.1007/s00114-017-1496-y.

Lü, J. (2003). A new oviraptorosaurid (Theropoda: Oviraptorosauria) from the Late Cretaceous of southern China. *J. Vertebr. Paleontol.* 22, 871–875. doi:10.1671/0272-4634(2002)022[0871:ANOTOF]2.0.CO;2.

Lü, J., and Brusatte, S. L. (2015). A large, short-armed, winged dromaeosaurid (Dinosauria: Theropoda) from the Early Cretaceous of China and its implications for feather evolution. *Sci. Rep.* 5, 11775. doi:10.1038/srep11775.

Lü, J. C., Tomida, Y., Azuma, Y., Dong, Z. M., and Lee, Y.-N. (2004). New Oviraptorid Dinosaur (Dinosauria: Oviraptorosauria) from the Nemegt Formation of Southwestern Mongolia. *Bull Natn Sci Mus Tokyo Ser. C* 31, 51.

Lü, J. C., Tomida, Y., Azuma, Y., Dong, Z. M., and Lee, Y.-N. (2005a). *Nemegtomaia* gen. nov., a replacement name for the oviraptorosaurian dinosaur *Nemegtia* Lü et al., 2004, a preoccupied name. *Bull Natn Sci Mus Tokyo Ser. C* 31, 51.

Lü, J. C., Xu, L., Zhang, X. L., Ji, Q., Jia, S. H., Hu, W. Y., et al. (2007). New dromaeosaurid dinosaur from the Late Cretaceous Qiupa Formation of Luanchuan area, western Henan, China. *Geol. Bull. China* 26, 777–786.

Lü, J., Chen, R., Brusatte, S. L., Zhu, Y., and Shen, C. (2016). A Late Cretaceous diversification of Asian oviraptorid dinosaurs: evidence from a new species preserved in an unusual posture. *Sci. Rep.* 6, 35780. doi:10.1038/srep35780.

Lü, J., Currie, P. J., Xu, L., Zhang, X., Pu, H., and Jia, S. (2013a). Chicken-sized oviraptorid dinosaurs from central China and their ontogenetic implications. *Naturwissenschaften*. doi:10.1007/s00114-012-1007-0.

Lü, J., Huang, D., and Qiu, L. (2005b). "The pectoral girdle and the forelimb of Heyuannia (Dinosauria: Oviraptorosauria)," in *Carnivorous Dinosaurs*, ed. K. Carpenter (Bloomington: Indiana University Press), 256–273.

Lü, J., Yi, L., Zhong, H., and Wei, X. (2013b). A New Oviraptorosaur (Dinosauria: Oviraptorosauria) from the Late Cretaceous of Southern China and Its Paleoecological Implications. *PLoS ONE* 8. doi:10.1371/journal.pone.0080557.

Makovicky, P. J., and Sues, H.-D. (1998). Anatomy and phylogenetic relationships of the theropod dinosaur Microvenator celer from the Lower Cretaceous of Montana. American Museum novitates; no. 3240.

Motta, M. J., Agnolín, F. L., Brissón Egli, F., and Novas, F. E. (2020). New theropod dinosaur from the Upper Cretaceous of Patagonia sheds light on the paravian radiation in Gondwana. *Sci. Nat.* 107, 24. doi:10.1007/s00114-020-01682-1.

Norell, M. A., Makovicky, P., and Clark, J. M. (1997). A *Velociraptor* wishbone. *Nature* 389, 447–447. doi:10.1038/38918.

Novas, F. E., Egli, F. B., Agnolin, F. L., Gianechini, F. A., and Cerda, I. (2018). Postcranial osteology of a new specimen of*Buitreraptor gonzalezorum* (Theropoda, Unenlagiidae). *Cretac. Res.* 83, 127–167. doi:10.1016/j.cretres.2017.06.003.

Novas, F. E., and Puerta, P. F. (1997). New evidence concerning avian origins from the Late Cretaceous of Patagonia. *Nature* 387, 390–392.

Ősi, A., Apesteguía, S., and Kowalewski, M. (2010). Non-avian theropod dinosaurs from the early Late Cretaceous of central Europe. *Cretac. Res.* 31, 304–320. doi:10.1016/j.cretres.2010.01.001.

Osmólska, H., Currie, P. J., and Barsbold, R. (2007). "Oviraptorosauria," in *The dinosauria* (Univ of California Press).

Ostrom, J. H. (1969). *A new theropod dinosaur from the Lower Cretaceous of Montana*. Peabody Museum of Natural History.

Ostrom, J. H. (1974). The pectoral girdle and forelimb function of *Deinonychus* (Reptilia: Saurischia): a correction.

Parsons, W. L., and Parsons, K. M. (2009). Further descriptions of the osteology of *Deinonychus antirrhopus* (Saurischia, Theropoda). *Bull. Buffalo Soc. Nat. Sci.* 38, 43–54.

Pei, R., Li, Q., Meng, Q., Norell, M. A., and Gao, K.-Q. (2017). New specimens of *Anchiornis huxleyi* (Theropoda: Paraves) from the Late Jurassic of northeastern China. *Bull. Am. Mus. Nat. Hist.* 2017, 1–67. doi:10.1206/0003-0090-411.1.1.

Perle, A., Norell, M., and Clark, J. M. (1999). *A new maniraptoran theropod,* Achillobator giganticus *(Dromaeosauridae), from the Upper Cretaceous of Burkhant, Mongolia*. Geology and Mineralogy Chair, National University of Mongolia.

Poust, A. W. (2014). Description and ontogenetic assessment of a new Jehol microraptorine.

Poust, A. W., Gao, C., Varricchio, D. J., Wu, J., and Zhang, F. (2020). A new microraptorine theropod from the Jehol Biota and growth in early dromaeosaurids. *Anat. Rec.*, 1–25. doi:10.1002/ar.24343.

Qiu, R., Wang, X., Wang, Q., Li, N., Zhang, J., and Ma, Y. (2019). A new caudipterid from the Lower Cretaceous of China with information on the evolution of the manus of Oviraptorosauria. *Sci. Rep.* 9, 1–10. doi:10.1038/s41598-019-42547-6.

Russell, D. A., and Dong, Z.-M. (1993). A nearly complete skeleton of a new troodontid dinosaur from the Early Cretaceous of the Ordos Basin, Inner Mongolia, People's Republic of China. *Can. J. Earth Sci.* 30, 2163–2173.

Shen, C., Lue, J., Liu, S., Kundrat, M., Brusatte, S. L., and Gao, H. (2017). A new troodontid dinosaur from the Lower Cretaceous Yixian Formation of Liaoning Province, China. *Acta Geol. Sin.-Engl. Ed.* 91, 763–780. doi:10.1111/1755-6724.13307.

Tsuihiji, T., Barsbold, R., Watabe, M., Tsogtbaatar, K., Chinzorig, T., Fujiyama, Y., et al. (2014). An exquisitely preserved troodontid theropod with new information on the palatal structure from the Upper Cretaceous of Mongolia. *Naturwissenschaften* 101, 131–142. doi:10.1007/s00114-014-1143-9.

Wang, M., O'Connor, J. K., Xu, X., and Zhou, Z. (2019). A new Jurassic scansoriopterygid and the loss of membranous wings in theropod dinosaurs. *Nature* 569, 256–259. doi:10.1038/s41586-019-1137-z.

Wei, X., Pu, H., Xu, L., Liu, D., and Lü, J. (2013). A new oviraptorid dinosaur (Theropoda: Oviraptorosauria) from the Late Cretaceous of Jiangxi Province, southern China. *Acta Geol. Sin. Ed.* 87, 899–904. doi:10.1111/1755-6724.12098.

Xu, X., Choiniere, J. N., Pittman, M. D., Tan, Q., Xiao, D., Li, Z., et al. (2010). A new dromaeosaurid (Dinosauria: Theropoda) from the Upper Cretaceous Wulansuhai formation of inner Mongolia, China. *Zootaxa* 2403, 1–9.

Xu, X., Currie, P., Pittman, M., Xing, L., Meng, Q., Lü, J., et al. (2017a). Mosaic evolution in an asymmetrically feathered troodontid dinosaur with transitional features. *Nat. Commun.* 8, 14972. doi:10.1038/ncomms14972.

Xu, X., Norell, M. A., Kuang, X., Wang, X., Zhao, Q., and Jia, C. (2004). Basal tyrannosauroids from China and evidence for protofeathers in tyrannosauroids. *Nature* 431, 680–684. doi:10.1038/nature02855.

Xu, X., Qin, Z., and Palasiatica, V. (2017b). A new tiny dromaeosaurid dinosaur from the Lower Cretaceous Jehol Group of western Liaoning and niche differentiation among the Jehol dromaeosaurids. *Vertebr. Palasiat.* 55, 129–144.

Xu, X., Sullivan, C., and Wang, S. (2013a). The systematic position of the enigmatic theropod dinosaur Yixianosaurus longimanus. *Vertebr. Palasiat.* 51, 169–183.

Xu, X., Tan, Q., Sullivan, C., Han, F., and Xiao, D. (2011a). A Short-Armed Troodontid Dinosaur from the Upper Cretaceous of Inner Mongolia and Its Implications for Troodontid Evolution. *PLoS ONE* 6. doi:10.1371/journal.pone.0022916.

Xu, X., Tan, Q., Wang, J., Zhao, X., and Tan, L. (2007). A gigantic bird-like dinosaur from the Late Cretaceous of China. *Nature* 447, 844–847. doi:10.1038/nature05849.

Xu, X., Tan, Q., Wang, S., Sullivan, C., Hone, W. E. D., Han, F., et al. (2013b). A new oviraptorid from the Upper Cretaceous of Nei Mongol, China, and its stratigraphic implications. *Vertebr. Palasiat.* 52, 85–101.

Xu, X., Wang, X., and Wu, X. (1999). A dromaeosaurid dinosaur with a filamentous integument from the Yixian Formation of China. *Nature* 401, 262–266. doi:10.1038/45769.

Xu, X., and Wang, X.-L. (2003). A new maniraptoran dinosaur from the Early Cretaceous Yixian Formation of western Liaoning. *Vertebr. Palasiat.* 41, 195–208.

Xu, X., You, H., Du, K., and Han, F. (2011b). An *Archaeopteryx*-like theropod from China and the origin of Avialae. *Nature* 475, 465–470. doi:10.1038/nature10288.

Xu, X., Zhao, Q., Norell, M., Sullivan, C., Hone, D., Erickson, G., et al. (2009). A new feathered maniraptoran dinosaur fossil that fills a morphological gap in avian origin. *Chin. Sci. Bull.* 54, 430–435. doi:10.1007/s11434-009-0009-6.

Xu, X., Zheng, X., Sullivan, C., Wang, X., Xing, L., Wang, Y., et al. (2015). A bizarre Jurassic maniraptoran theropod with preserved evidence of membranous wings. *Nature* 521, 70–73. doi:10.1038/nature14423.

Xu, X., Zhou, Z., Wang, X., Kuang, X., Zhang, F., and Du, X. (2003). Four-winged dinosaurs from China. *Nature* 421, 335–340. doi:10.1038/nature01342.

Zhang, F., Zhou, Z., Xu, X., and Wang, X. (2002). A juvenile coelurosaurian theropod from China indicates arboreal habits. *Naturwissenschaften* 89, 394–398. doi:10.1007/s00114-002-0353-8.

Zhang, F.-C., Zhou, Z.-H., Xu, X., Wang, X.-L., and Sullivan, C. (2008). A bizarre Jurassic maniraptoran from China with elongate ribbon-like feathers. *Nature* 455, 1105–1108. doi:10.1038/npre.2008.2326.1.

Zheng, X., Xu, X., You, H., Zhao, Q., and Dong, Z. (2010). A short-armed dromaeosaurid from the Jehol Group of China with implications for early dromaeosaurid evolution. *Proc. R. Soc. B Biol. Sci.* 277, 211–217. doi:10.1098/rspb.2009.1178.

Zhou, Z., Wang, X., Zhang, F., and Xu, X. (2000). Important features of *Caudipteryx* evidence from two nearly complete new specimens. *Vertebr. Palasiat.*

Zhou, Z.-H., and Wang, X.-L. (2000). A new species of *Caudipteryx* from the Yixian Formation of Liaoning northeast of China. *Vertebr. Palasiat.*
